# High-Glucose Environment Promotes Radioresistance in Colorectal Cancer via SERPINE1-Enhanced DNA Repair and Anti-Apoptosis

**DOI:** 10.7150/ijms.130794

**Published:** 2026-09-18

**Authors:** Chien-Chih Ke, Yu-Chen Zhang, Po-Jen Wang, Chen-Yu Wang, Shih-Hsun Kuo, Chia-Yang Li, Ming-Yii Huang, Ya-Ju Hsieh

**Affiliations:** 1 Department of Medical Imaging and Radiological Sciences, Kaohsiung Medical University, Kaohsiung, Taiwan.; 2 Department of Medical Research, Kaohsiung Medical University Hospital, Kaohsiung, Taiwan.; 3 Drug Development and Value Creation Research Center, Kaohsiung Medical University, Kaohsiung, Taiwan.; 4 Department of Radiation Oncology, Kaohsiung Medical University Hospital, Kaohsiung Medical University, Kaohsiung, Taiwan.; 5 Graduate Institute of Medicine, Kaohsiung Medical University, Kaohsiung, Taiwan.; 6 Department of Radiation Oncology, School of Medicine, College of Medicine, Kaohsiung Medical University, Kaohsiung, Taiwan.; 7 Center for Cancer Research, Kaohsiung Medical University, Kaohsiung, Taiwan.

**Keywords:** colorectal cancer, hyperglycemia, radioresistance, SERPINE1, DNA repair, cancer stem cells

## Abstract

**Background:**

Patients with colorectal cancer (CRC) and comorbid hyperglycemia often exhibit poor responses to preoperative concurrent chemoradiotherapy (CCRT), leading to unfavorable clinical outcomes. However, how hyperglycemia contributes to CRC radioresistance remains incompletely understood. This study aimed to elucidate the molecular mechanisms by which high-glucose exposure promotes radioresistance in CRC.

**Methods:**

CRC cell lines (SW480 and HCT116) were cultured under normal-glucose (5 mM), high-glucose (25 mM), or mannitol osmotic control conditions to assess malignant behaviors and radiosensitivity. Relative miRNA expression changes from pooled plasma exosomal miRNA profiling were integrated with TargetScan, miRDB, GeneCards, and TCGA analyses to screen for candidate mediators. Candidate SERPINE1-regulating miRNAs were validated using mimic/inhibitor assays. The functional role of SERPINE1 was validated using siRNA knockdown and plasmid overexpression assays, followed by assays of clonogenic survival, apoptosis, γ-H2AX foci kinetics, and DNA repair-associated protein expression.

**Results:**

High-glucose culture significantly enhanced CRC cell proliferation, migration, invasion, epithelial-mesenchymal transition, cancer stemness, and clonogenic radioresistance, whereas mannitol did not reproduce these effects. Integrated exploratory exosomal miRNA and bioinformatic analyses identified SERPINE1 as a candidate mediator associated with hyperglycemia, radioresistance, and poor prognosis. Selected hyperglycemia-associated miRNAs functionally modulated SERPINE1 protein expression in CRC cells. We confirmed that high glucose upregulates SERPINE1 expression *in vitro*. Mechanistically, SERPINE1 knockdown suppressed malignant phenotypes, reduced stemness-associated traits, enhanced irradiation-induced apoptosis, increased residual γ-H2AX foci, and attenuated DNA repair-associated protein responses. Conversely, SERPINE1 overexpression promoted aggressive phenotypes, reduced apoptotic signaling, accelerated γ-H2AX foci resolution, and enhanced radioresistance.

**Conclusion:**

High-glucose exposure promotes CRC malignancy and radioresistance, at least in part, through SERPINE1 upregulation. SERPINE1 contributes to radioresistance by attenuating apoptosis and facilitating radiation-induced DNA damage resolution. These findings support SERPINE1 as a key mechanistic mediator linking high-glucose exposure to radioresistance in CRC cell models. Because the exosomal miRNA profiling was performed using pooled plasma samples, the resulting miRNA findings should be regarded as exploratory and do not constitute a validated biomarker signature. The potential clinical relevance of SERPINE1 requires further validation in independent patient cohorts and physiologically relevant *in vivo* models.

## Introduction

Colorectal cancer (CRC) remains a major global health burden, ranking as the third most commonly diagnosed cancer and the second leading cause of cancer-related deaths worldwide 1. For patients with locally advanced rectal cancer, preoperative concurrent chemoradiotherapy (CCRT) followed by surgery is the standard therapeutic strategy 2, 3. This multimodal approach aims to downstage the tumor, increase the rate of sphincter preservation, and improve local control. However, the therapeutic response to CCRT varies significantly among individuals. Approximately 30-40% of patients exhibit resistance to radiotherapy, resulting in poor tumor regression and subjecting patients to unnecessary treatment toxicity without clinical benefit 3. Therefore, identifying the factors contributing to radioresistance is crucial for improving therapeutic outcomes.

Metabolic disorders, particularly hyperglycemia and diabetes mellitus, have emerged as significant risk factors for cancer progression and treatment failure 4-6. Epidemiological studies indicate that a substantial proportion of CRC patients present with comorbid hyperglycemia, a condition associated with higher rates of recurrence, metastasis, and mortality compared to normoglycemic patients 7. Importantly, clinical evidence suggests that diabetic CRC patients respond poorly to neoadjuvant CCRT, exhibiting lower rates of pathologic complete response (pCR) and worse overall survival 7-10. Studies have also demonstrated the strong link between hyperglycemia and cancer aggressiveness 11. Elevated glucose levels have been shown to directly stimulate cancer cell proliferation and accelerate cell division, leading to rapid tumor growth 12, 13. Furthermore, a hyperglycemic microenvironment promotes a more malignant phenotype by enhancing cell migration and invasive potential, often facilitating the epithelial-mesenchymal transition (EMT) process, a key step in tumor metastasis 14, 15. Crucially, high glucose conditions contribute to therapeutic failure by conferring resistance to apoptosis and chemotherapy, allowing cancer cells to survive under stress that would normally induce cell death 16, 17. While high glucose levels are known to enhance these aggressive behaviors, the precise molecular mechanisms by which a hyperglycemic microenvironment confers radioresistance in CRC remain incompletely understood.

Although the association between hyperglycemia and therapy-refractory CRC is increasingly recognized, the key molecular mediators that couple metabolic dysregulation to radioresistance remain incompletely defined. To uncover clinically relevant drivers, we employed an integrated approach combining patient plasma exosomal miRNA profiling with bioinformatic analysis to narrow down potential modulating targets. Among the candidates identified through this screening strategy, SERPINE1 (also known as Plasminogen Activator Inhibitor-1, PAI-1) emerged as a promising candidate mediator. Functionally, SERPINE1 is a primary inhibitor of tissue plasminogen activator (tPA) and urokinase plasminogen activator (uPA), traditionally known for its role in fibrinolysis and thrombosis 18, 19. Recent studies have implicated aberrant SERPINE1 expression in various malignancies, linking it to tumor aggressiveness, EMT, and poor prognosis 20-23. However, whether and how SERPINE1 links hyperglycemia to radiation resistance in CRC has not been fully elucidated.

In this study, we hypothesized that a high-glucose microenvironment upregulates SERPINE1 expression, which in turn orchestrates a radioresistant and aggressive phenotype in CRC cells. Consequently, we investigated the impact of high glucose on cell malignancy, EMT, and cancer stemness, and systematically explored the functional role of SERPINE1 in modulating DNA repair mechanisms and apoptosis following radiation.

## Materials and Methods

### Cell culture

The human CRC cell lines SW480 and HCT116 were obtained from the American Type Culture Collection (ATCC). Cells were cultured in Dulbecco's Modified Eagle Medium (DMEM; Gibco, ThermoFisher) supplemented with 10% Fetal Bovine Serum (FBS; Seradigm, VWR), 1% Non-Essential Amino Acids (NEAA), 1% Sodium Pyruvate, and 1% Penicillin/Streptomycin/Amphotericin (PSA; Capricorn-scientific). To simulate hyperglycemic conditions *in vitro*, cells were maintained in DMEM containing either 5 mM glucose (normal glucose, NG) or 25 mM glucose (high glucose, HG). An osmotic control condition was included by supplementing 5 mM glucose medium with 20 mM mannitol, thereby matching the osmolar contribution of the 25 mM glucose condition. Cells cultured under the aforementioned conditions were incubated at 37 °C in a humidified atmosphere with 5% CO_2_.

### Plasma exosomal miRNA isolation from CRC patients

The study protocol involving human participants was reviewed and approved by the Institutional Review Board of Kaohsiung Medical University Hospital (Protocol No: KMUHIRB-E(I)-20210384). Informed consent was obtained from all participants. A total of 10 CRC patients were enrolled and categorized into two groups based on their glycemic status: the hyperglycemia group (n = 4), defined as patients with a history of diabetes mellitus and HbA1c levels ≥ 6%, and the normoglycemia group (n = 6), defined as patients with no history of diabetes and HbA1c levels < 6%. Because the patient blood samples were collected for various sub-projects to conduct different research analyses, the plasma volume allocated to this study was limited. To obtain sufficient RNA for exosomal miRNA profiling, plasma samples within each group were pooled to generate two composite samples: one pooled sample for the hyperglycemia group and one pooled sample for the normoglycemia group. The exosomal yield from an individual's allocated volume was technically insufficient to meet the minimum RNA input required for small RNA Next-Generation Sequencing (NGS). Therefore, this analysis was intended for exploratory candidate discovery rather than individual-level differential expression testing. Exosomes were subsequently isolated from these pooled plasma samples using the Total Exosome Isolation Kit (from plasma) (Thermo Fisher Scientific, Waltham, MA, USA) according to the manufacturer's instructions.

### miRNA sequencing and bioinformatics analysis

Total RNA extraction and small RNA Next-Generation Sequencing (NGS) were conducted by Genomics BioSci & Tech Co., Ltd. (New Taipei City, Taiwan). The sequencing data from the two pooled composite samples were analyzed to identify relative miRNA expression changes between the hyperglycemia and normoglycemia groups. miRNAs showing lower expression in the pooled hyperglycemia sample were selected for downstream target prediction. Potential target genes of these miRNAs were predicted using miRDB and TargetScan databases. To identify candidate genes associated with radioresistance in hyperglycemic CRC, these targets were intersected with gene sets related to "Colorectal Cancer," "Hyperglycemia," and "Radioresistance" retrieved from the GeneCards database. The clinical prognostic value of candidate genes was evaluated using the TCGA CRC dataset via the DriverDBv4 database.

### Cell transfection

For SERPINE1 loss- and gain-of-function experiments, cells were maintained under high-glucose conditions during transfection and subsequent functional assays. For gene knockdown, SW480 cells were transfected with small interfering RNA (siRNA) targeting *SERPINE1* mRNA or a negative control (NC) siRNA (GenePharma) using DharmaFECT Transfection Reagents (Dharmacon). For gene overexpression, HCT116 cells were transfected with a *SERPINE1* ORF cDNA clone expression plasmid or a pCMV3-untagged NC vector (Sino Biological). For miRNA functional validation, synthetic hsa-miRNA mimics and inhibitors were purchased from Bio-Genesis Technologies, Inc. Five miRNAs predicted to potentially regulate SERPINE1 were selected for further validation, including hsa-miR-483-5p, hsa-miR-6873-3p, hsa-miR-4771, hsa-miR-7111-3p, and hsa-miR-3652. SW480 and HCT116 cells were transiently transfected with the indicated miRNA mimics or corresponding miRNA inhibitors using DharmaFECT Transfection Reagents according to the manufacturer's instructions. NC mimic and inhibitor oligonucleotides were used as controls. The sequences of miRNA mimics and inhibitors are listed in Table S1. Transfections were performed according to the manufacturer's protocol. Briefly, cells were seeded in 6-well plates and transfected when reaching appropriate confluency. The medium was replaced with complete medium 24 h after transfection. At 48 h after transfection, cells were harvested for subsequent functional assays or Western blot analysis to confirm SERPINE1 knockdown, SERPINE1 overexpression, or miRNA-mediated changes in SERPINE1 protein expression.

### Cell viability assay

Cell proliferation was assessed using the MTT assay. Cells were seeded into 96-well plates (1,000 cells/well) and allowed to adhere overnight. At indicated time points (1-5 days), cells were incubated with 3-(4,5-Dimethylthiazol-2-yl)-2,5-diphenyltetrazolium bromide (MTT, 5 mg/mL) for 3 h at 37 °C. The formazan crystals were dissolved in dimethyl sulfoxide (DMSO), and absorbance was measured at 570 nm using a microplate reader (Bio-Rad).

### Migration and invasion assays

For the wound-healing assay, cells were seeded in 24-well plates and grown to confluence. A scratch was created using a sterile pipette tip, and cells were washed with PBS to remove debris. Wound closure was monitored and photographed after 24 h. Transwell assays were performed using Transwell inserts (Corning). For invasion, the upper chamber was pre-coated with Matrigel (Corning) before cell seeding. The lower chamber contained complete medium with 10% FBS as a chemoattractant. After 24 h, cells on the upper surface were removed, and migrated or invaded cells on the lower surface were fixed, stained with crystal violet, and counted under a microscope.

### Sphere formation assay

To evaluate cancer stemness, cells were dissociated and seeded at a density of 2,000 cells/well in ultra-low attachment 6-well plates (Corning). Cells were cultured in serum-free sphere medium supplemented with B27, 20 ng/mL hEGF, and 5 ng/mL bFGF. Tumor spheres were allowed to form for 7-14 days, and sphere number and size were analyzed.

### Radiation clonogenic assay

For the colony formation assay, cells were seeded in 6-well plates and incubated for 14 days to form colonies. For the radiation clonogenic assay, seeded cells were exposed to single doses of X-ray radiation (0, 2, or 6 Gy) using a linear accelerator (Elekta Axesse™, 6 MV). After irradiation, cells were cultured for 14 days. Colonies (≥ 50 cells per colony) were fixed with methanol, stained with 1% crystal violet, and counted. The surviving fraction (SF) was calculated as: SF = (Mean number of colonies) / (Number of seeded cells × Plating Efficiency).

### γ-H2AX immunofluorescence staining and foci quantification

To evaluate radiation-induced DNA damage resolution, γ-H2AX immunofluorescence staining was performed. SERPINE1-knockdown SW480 cells and SERPINE1-overexpressing HCT116 cells, together with their corresponding control cells, were seeded on sterile coverslips and exposed to 6 Gy irradiation. Cells were fixed at baseline and at 12 and 24 h after irradiation. After fixation with 4% paraformaldehyde, cells were permeabilized with Triton X-100 and blocked with bovine serum albumin-containing blocking buffer. Cells were then incubated with an anti-γ-H2AX primary antibody (ABclonal Technology), followed by an appropriate fluorescence-conjugated secondary antibody (ABclonal Technology). Nuclei were counterstained with DAPI. Fluorescence images were acquired using a fluorescence microscope, and γ-H2AX foci were quantified as the number of foci per nucleus. Three independent experiments were performed, and multiple random fields were analyzed for each condition.

### Western blot analysis

Total protein was extracted using RIPA lysis buffer containing protease inhibitors. Protein concentration was determined using the Bradford assay. Equal amounts of protein samples (30-50 µg) were separated by SDS-PAGE and transferred onto PVDF membranes. Membranes were blocked with 5% BSA and incubated overnight at 4 °C with primary antibodies against E-cadherin, Vimentin, Snail, Twist, OCT4, Sox2, ALDH1, cleaved-PARP, cleaved-Caspase-3, BAX, BCL2, RAD50, NBS1, MRE11, Ku70, Ku80, SERPINE1, and β-actin (as loading control) (ABclonal Technology). After incubation with secondary antibodies, protein bands were visualized using an ECL detection system (Advansta) and quantified using ImageJ software.

### Statistical analysis

All experiments were performed in at least triplicate. Data are presented as mean ± standard deviation (SD). Statistical analysis was performed using GraphPad Prism 6.0 software. For comparisons between two groups, Student's t-test was used. For comparisons among three or more groups, one-way ANOVA followed by Tukey's or Dunnett's post hoc test was performed. For experiments involving two independent variables, such as treatment group and time point, two-way ANOVA followed by Tukey's post hoc test was used. A *p*-value < 0.05 was considered statistically significant.

## Results

### High glucose environment promotes CRC cell proliferation and motility

To evaluate the impact of hyperglycemia on CRC progression, we cultured SW480 and HCT116 cells in normal glucose (NG, 5 mM) or high glucose (HG, 25 mM) conditions. The MTT assay indicated that both cells in the HG group exhibited significantly higher proliferation rates compared to the NG group (Figure 1A). Similarly, wound-healing and transwell invasion assays demonstrated that high glucose markedly accelerated cell migration and invasion (Figure 1B, C). To understand the molecular basis of this aggressive phenotype, we examined EMT markers. Western blot analysis revealed that HG treatment suppressed the epithelial marker E-cadherin and upregulated the mesenchymal marker Vimentin, along with EMT-inducing transcription factors Snail and Twist (Figure 1D, E). These data suggest that HG promotes malignant behaviors in CRC cells in association with proliferation and EMT-related reprogramming.

### High glucose enhances CRC cancer stemness and clonogenic radioresistance

EMT programs are frequently coupled with acquisition of cancer stem cells (CSC)-like features, which are closely associated with tumor recurrence. Therefore, we investigated the effect of high glucose on stemness. Sphere formation assays demonstrated that HG significantly enhanced sphere-forming capacity, indicating increased self-renewal potential (Figure 2C, D). Consistently, HG increased CSC-associated protein expression, including OCT4 and ALDH1 (Figure 2A, B), supporting activation of stemness-related programs. These results indicate that hyperglycemic culture conditions promote CSC-like traits in CRC cells, providing a mechanistic bridge between HG exposure and increased malignant potential.

We next evaluated whether HG impacts intrinsic radiosensitivity by performing clonogenic survival assays following irradiation at 0, 2, and 6 Gy. Under HG conditions, SW480 cells displayed greater clonogenic survival than HCT116 cells, indicating a higher baseline radioresistance in SW480 cells (Figure 3A). In SW480 cells, the surviving fractions were significantly higher under HG conditions than under NG conditions at both 2 Gy and 6 Gy. In HCT116 cells, however, a significant increase was observed at 6 Gy but not at 2 Gy (Figure 3B, C). Collectively, these results indicate that HG not only enhances malignant phenotypes and CSC-like traits but also promotes clonogenic radioresistance in CRC cells.

### High glucose-induced CRC malignant phenotypes are independent of hyperosmolarity

To rule out the possibility that the observed malignant phenotypes were merely artifacts of hyperosmotic stress induced by high glucose levels, an osmotic control group (5 mM glucose supplemented with 20 mM mannitol) was included. Crucially, the addition of mannitol did not alter cell proliferation or migration capacity compared to the 5 mM normal glucose group (Figure S1). Furthermore, the 25 mM glucose-induced expression of EMT markers (downregulation of E-cadherin; upregulation of Vimentin, Snail, and Twist) and the stemness marker OCT4 were not observed in the osmotic control group (Figure S2). Most importantly, clonogenic survival assays confirmed that enhanced radioresistance following 2 to 6 Gy irradiation was specifically driven by glucose rather than osmolarity, as the surviving fractions in the 5 mM + mannitol group remained comparable to those in the 5 mM glucose group (Figure S3). Collectively, these data confirm that glucose specifically promotes EMT, stemness, and radioresistance in CRC cells.

### Integrated bioinformatics analysis identifies SERPINE1 as a potential key mediator

Having established that HG promotes malignant behaviors, CSC-like traits, and radioresistance, we next sought to identify candidate mediators that may link hyperglycemia to therapy-refractory CRC. The plasma exosomes from CRC patients were collected and stratified into a hyperglycemia group (n = 4; HbA1c ≥ 6%) and a normoglycemia group (n = 6; HbA1c < 6%). Samples were then pooled within each group and subjected to miRNA sequencing. Because the plasma samples were pooled within each group, this analysis was used as an exploratory, hypothesis-generating screening approach rather than as a definitive individual-level differential expression analysis. Comparative miRNA profiling identified 12 miRNAs with higher expression and 17 miRNAs with lower expression in the pooled hyperglycemia sample compared with the pooled normoglycemia sample, using a threshold of |log2 fold change (log2FC)| ≥ 1. Based on this exploratory result, we hypothesized that downregulation of these 17 miRNAs may lead to derepression of specific target genes that contribute to hyperglycemia-associated CRC malignancy and radioresistance. To test this, we predicted the target genes of these 17 downregulated miRNAs using miRDB and TargetScan databases, yielding 4,527 potential targets (File S1). Concurrently, we retrieved 555 genes associated with "Colorectal Cancer," "Hyperglycemia," and "Radioresistance" from the GeneCards database (File S2). Intersecting these datasets narrowed the list down to 152 candidate genes (Figure S4A). Bioinformatic survival analysis using the TCGA dataset prioritized SERPINE1, as its high expression was significantly associated with poor overall survival (Log-Rank p = 0.0023) and was elevated in tumor tissues compared to normal tissues. Consequently, SERPINE1 was selected as the key candidate for functional validation (Figure S4B).

### Functional validation of candidate SERPINE1-regulating miRNAs in CRC cells

After SERPINE1 was identified as a key candidate, we re-examined the TargetScan and miRDB prediction results to identify candidate SERPINE1-regulating miRNAs among the hyperglycemia-associated downregulated exosomal miRNAs. Five miRNAs—miR-483-5p, miR-3652, miR-4771, miR-6873-3p, and miR-7111-3p—were predicted as potential regulators of SERPINE1 and were therefore selected for mimic/inhibitor-based functional validation. Transfection with these miRNA mimics reduced SERPINE1 protein expression in both SW480 and HCT116 cells, with statistically significant suppression observed for most candidate miRNAs (Figure 4). Conversely, the corresponding miRNA inhibitors generally increased SERPINE1 expression, although the response varied among individual miRNAs and cell lines. Significant inhibitor-induced SERPINE1 upregulation was observed for miR-3652, miR-4771, and miR-6873-3p in SW480 cells (Figure 4A), and for miR-4771, miR-6873-3p, and miR-7111-3p in HCT116 cells (Figure 4B). Taken together, these data indicate that the selected hyperglycemia-associated miRNAs can functionally regulate SERPINE1 expression in CRC cells, supporting a possible miRNA-mediated regulatory mechanism contributing to SERPINE1 upregulation.

### SERPINE1 promotes CRC cell proliferation, migration, and invasion

Given that SW480 cells exhibited relatively higher clonogenic radioresistance than HCT116 cells under high-glucose conditions, we used SW480 cells for SERPINE1 loss-of-function experiments and HCT116 cells for SERPINE1 gain-of-function experiments. Under high-glucose conditions, siRNA-mediated SERPINE1 knockdown in SW480 cells significantly reduced cell proliferation, delayed wound closure, and decreased Matrigel-based invasion compared with control siRNA-transfected cells (Figure 5A-C). These findings indicate that SERPINE1 silencing attenuates high glucose-associated malignant behaviors in radioresistant SW480 cells. As a complementary gain-of-function approach, SERPINE1 overexpression in HCT116 cells under high-glucose conditions enhanced proliferation, migration, and invasion compared with vector control cells (Figure 5D-F), supporting a pro-malignant role of SERPINE1 in the high-glucose context.

### SERPINE1 enhances CSC-like traits and stemness marker expression

We further evaluated stemness-related phenotypes following SERPINE1 perturbation. Under high-glucose conditions, SERPINE1 knockdown in SW480 cells reduced the expression of CSC-associated markers OCT4 and ALDH1, accompanied by a significant reduction in sphere-forming capacity (Figure 6A, C), indicating that SERPINE1 silencing attenuated high glucose-enhanced self-renewal potential. In contrast, SERPINE1 overexpression in HCT116 cells increased OCT4 and ALDH1 expression and markedly enhanced sphere formation (Figure 6B, D). Together, these findings suggest that elevated SERPINE1 expression promotes CSC-like properties and reinforces high glucose-associated stemness traits.

### SERPINE1 mediates radioresistance by modulating apoptosis

To test whether SERPINE1 contributes to CRC radioresistance, cell clonogenic survival assay following irradiation at 0, 2, and 6 Gy was performed in both cells after gain- and loss-of-function experiments. In SW480 cells cultured under high-glucose conditions, SERPINE1 knockdown decreased clonogenic survival compared with control siRNA, indicating increased radiosensitivity (Figure 7A). Conversely, SERPINE1 overexpression in HCT116 cells cultured under high-glucose conditions increased clonogenic survival compared with vector control cells, suggesting that elevated SERPINE1 expression enhances high glucose-associated radioresistance (Figure 7B).

Because clonogenic survival reflects long-term reproductive viability and may be influenced by apoptosis after irradiation, we next examined the expression of apoptosis-related proteins at 0, 4, and 12 h after irradiation under high-glucose conditions. In SW480 cells, SERPINE1 knockdown enhanced apoptotic signaling following irradiation, characterized by increased pro-apoptotic markers (e.g., cleaved caspase-3 and BAX) with reduced anti-apoptotic BCL2 (Figure 8A, B). In contrast, SERPINE1 overexpression in HCT116 cells attenuated irradiation-induced apoptotic responses, showing reduced cleaved caspase-3 and BAX expression together with increased BCL2 expression (Figure 8C, D). These findings suggest that SERPINE1 contributes to high glucose-associated radioresistance, at least in part, by suppressing apoptosis following irradiation.

### SERPINE1 is associated with enhanced DNA repair-related protein responses following irradiation

To assess whether SERPINE1 modulates radiation-induced DNA damage resolution, we first monitored the kinetics of γ-H2AX foci, a widely used surrogate marker of DNA double-strand breaks (DSB) responses, at 12 and 24 h following 6 Gy irradiation under high-glucose conditions. Immunofluorescence analysis showed that under basal conditions (without irradiation), γ-H2AX foci levels were low and did not differ significantly between control and SERPINE1-modulated cells. Following 6 Gy of irradiation, SERPINE1 knockdown in SW480 cells significantly increased the number of residual γ-H2AX foci per nucleus at both 12 and 24 h compared with the control siRNA group (Figure 9A), demonstrating a delayed resolution of radiation-induced DNA damage. Conversely, SERPINE1 overexpression in HCT116 cells under high-glucose conditions significantly reduced residual γ-H2AX foci at 12 and 24 h compared with the control vector group (Figure 9B), indicating a more efficient resolution of radiation-induced DNA damage.

To further examine whether these functional changes were accompanied by altered DNA repair-associated molecular responses, we assessed the expression profiles of key DNA DSB repair-related proteins at 0, 4, 12, and 24 h post-irradiation under high-glucose conditions. These included homologous recombination (HR)-associated MRN complex components, MRE11, RAD50, and NBS1, as well as non-homologous end joining (NHEJ)-associated proteins, Ku70 and Ku80. Consistent with the γ-H2AX foci kinetics, SERPINE1 knockdown in SW480 cells reduced baseline expression and/or post-irradiation induction of these repair-associated proteins (Figure 10). Conversely, SERPINE1 overexpression in HCT116 cells enhanced baseline and/or post-irradiation repair-associated protein responses (Figure 11). Taken together, these functional and molecular findings support that SERPINE1 contributes to radioresistance by facilitating the resolution of radiation-induced DNA damage, accompanied by enhanced DNA repair-associated protein responses following irradiation.

## Discussion

In this study, we addressed a critical clinical challenge: the poor therapeutic response of hyperglycemic CRC patients to preoperative CCRT. We provided evidence that a high-glucose microenvironment significantly enhances the malignant phenotype and radioresistance of CRC cells. Through an integrated bioinformatics approach starting from clinical plasma exosomal miRNAs, we identified SERPINE1 as a candidate molecular mediator linking metabolic dysregulation to therapy resistance. Our functional studies demonstrated that SERPINE1 upregulation under high-glucose conditions promotes EMT and cancer stemness, while facilitating DNA repair and suppressing apoptosis, ultimately conferring resistance to ionizing radiation.

While 25 mM glucose (approximately 450 mg/dL) exceeds the baseline diagnostic thresholds for diabetes, it represents a commonly used *in vitro* model of severe or poorly controlled hyperglycemic stress 8, 24, 25. Therefore, we used this concentration to investigate how extreme high-glucose exposure may influence CRC radioresistance. Importantly, the inclusion of a mannitol osmotic control showed that the enhanced malignant behaviors and radioresistant phenotypes were not reproduced by hyperosmolarity alone, supporting a glucose-associated effect. However, we acknowledge the inherent limitations of this static 2D model, which cannot fully capture dynamic glycemic fluctuations or complex interactions within the *in vivo* tumor microenvironment. Future studies using patient-derived organoids or *in vivo* diabetic murine models across a broader range of glucose concentrations will be necessary to further define the clinically relevant glucose-response relationship in CRC.

SERPINE1 is traditionally recognized as a primary inhibitor of tissue plasminogen activator (tPA) and urokinase plasminogen activator (uPA), regulating fibrinolysis and thrombosis 18. Beyond its hematological roles, emerging evidence implicates SERPINE1 in tumor progression, where it modulates cell adhesion, migration, and angiogenesis 20, 21, 26-28. Consistent with these previous studies, our bioinformatics analysis of the TCGA dataset showed that high SERPINE1 expression correlates with reduced overall survival in CRC patients (Figure S4). We extended these findings by demonstrating that SERPINE1 is not merely associated with prognosis but also functionally involved in CRC aggressiveness. The SERPINE1 loss- and gain-of-function experiments were performed under high-glucose conditions. SERPINE1 knockdown in the relatively radioresistant SW480 model attenuated high glucose-associated malignant and radioresistant phenotypes, whereas SERPINE1 overexpression in the relatively radiosensitive HCT116 model further enhanced aggressive traits, supporting the functional involvement of SERPINE1 in high glucose-associated CRC progression and radioresistance.

Our study further suggests that SERPINE1 contributes to radioresistance and CSC-like traits. Radiotherapy kills cancer cells primarily by inducing DNA DSBs. We observed that SERPINE1 knockdown compromised the expression of key DNA repair proteins involved in both HR (RAD50, MRE11, NBS1) and NHEJ (Ku70, Ku80) pathways following irradiation (Figure 10 and 11). This aligns with recent reports suggesting that SERPINE1 can translocate to the nucleus to facilitate DNA repair in response to radiation-induced damage 29. Furthermore, we established a link between SERPINE1 and cancer stemness. High glucose-induced SERPINE1 upregulation was accompanied by increased sphere-forming capacity and expression of stem cell markers OCT4 and ALDH1. Since CSC-like cells are often associated with radioresistance due to enhanced DNA repair capacity and anti-apoptotic signaling, our data suggest that SERPINE1 promotes radioresistance by maintaining a CSC-like state and enhancing DNA repair-associated responses.

The connection between hyperglycemia and cancer progression is well-documented, with high glucose known to accelerate proliferation and protect cells from apoptosis 8, 30-34. Our study provides a molecular explanation for this phenomenon in CRC. We found that SERPINE1 expression is responsive to glucose concentration, being significantly upregulated in a high-glucose environment. This upregulation may be mediated by oxidative stress or signaling pathways such as HIF-1α 8, which are activated under hyperglycemic conditions. By upregulating SERPINE1, the high-glucose microenvironment creates a pro-survival and radioresistant phenotype for cancer cells, enhancing their ability to repair radiation-induced DNA damage and survive therapeutic stress. This highlights the importance of strict glycemic control during cancer treatment and suggests that the metabolic state of the patient directly influences tumor biology.

Our findings suggest that SERPINE1 may represent a potential therapeutic target for overcoming radioresistance, particularly in hyperglycemia-associated CRC. Targeting SERPINE1, either through RNA interference or pharmacological inhibition, may provide a strategy to enhance radiosensitivity; however, its therapeutic potential requires further validation in physiologically relevant *in vivo* models. Several limitations of this study should also be acknowledged. First, the initial plasma exosomal miRNA sequencing relied on pooled samples from a small CRC cohort to obtain sufficient RNA for sequencing. While useful for exploratory profiling, this pooling strategy precludes assessment of inter-individual variability and limits robust statistical inference of differential miRNA expression at the individual-patient level. Furthermore, because the plasma samples allocated to this study were exhausted during the analysis, we were unable to validate the miRNA-SERPINE1 regulatory axis at the individual-patient level within this cohort. Consequently, these pooled exosomal miRNA findings should be regarded as strictly exploratory and hypothesis-generating, and the potential value of SERPINE1 as a biomarker candidate requires validation in independent clinical cohorts before any clinical application. Second, SERPINE1 prioritization was supported by bioinformatic and TCGA-based prognostic analyses, whereas direct validation of the candidate miRNAs and SERPINE1 expression in individual patient plasma samples, matched tumor tissues, or independent clinical cohorts was not performed in the present study. Third, although the miRNA mimic/inhibitor experiments support functional regulation of SERPINE1 by selected candidate miRNAs, direct interaction with the SERPINE1 3′UTR remains to be confirmed by luciferase reporter assays. Finally, the functional validation in this study was primarily performed in cell-line models; therefore, these findings do not yet establish the *in vivo* therapeutic efficacy of SERPINE1 inhibition. Future studies using patient-derived organoids and physiologically relevant hyperglycemic murine CRC models, such as xenograft or orthotopic tumor models, will be required to determine whether SERPINE1 knockdown or pharmacological inhibition can enhance tumor radiosensitivity *in vivo*. In addition, larger independent clinical cohorts with patient-level plasma exosomal and matched tumor tissue samples will be required to validate the candidate miRNA-SERPINE1 axis and determine its association with CCRT response in hyperglycemia-associated CRC.

## Conclusion

In conclusion, our findings identify SERPINE1 as a key mechanistic mediator of high-glucose-associated radioresistance in CRC cell models. High-glucose exposure upregulated SERPINE1, which promoted radioresistance through reduced apoptotic signaling and facilitated resolution of radiation-induced DNA damage. The exosomal miRNA findings were derived from pooled plasma samples and should therefore be regarded as exploratory rather than as a validated biomarker signature. Accordingly, the potential clinical relevance of SERPINE1 warrants further validation in independent patient cohorts and physiologically relevant *in vivo* models.

## Supplementary Material

Supplementary figures and table.

## Figures and Tables

**Figure 1:**
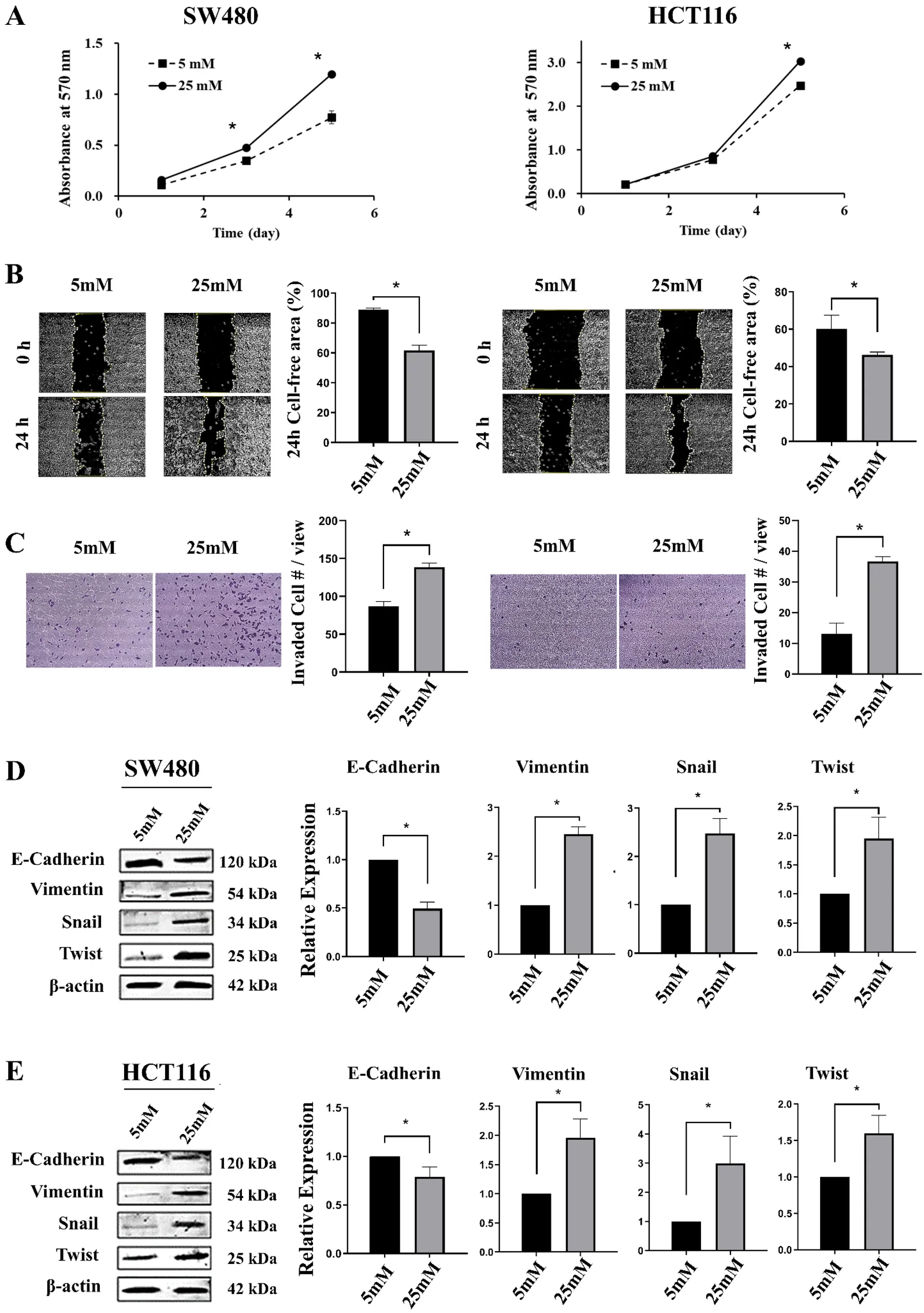
** High glucose environment promotes malignant phenotypes and EMT in CRC cells.** SW480 and HCT116 cells were cultured under normal glucose (5 mM) or high glucose (25 mM) conditions. **(A)** Cell proliferation was assessed using the MTT assay after cultured for 1, 3 and 5 days. **(B)** Cell migration was evaluated by wound-healing assay. Representative images (0 h and 24 h) and quantification of the cell-free area are shown. **(C)** Cell invasion was assessed by transwell assay with Matrigel. Representative images and quantification of invaded cells are presented. **(D, E)** Western blot analysis and quantification of EMT-related markers (E-cadherin, Vimentin, Snail, and Twist) in SW480 **(D)** and HCT116 **(E)** cells. β-actin served as a loading control. Data are expressed as mean ± SD from at least three independent experiments. For MTT cell proliferation, statistical significance was determined using two-way ANOVA followed by Tukey's post hoc test. For wound-healing, invasion, and Western blot quantification, statistical significance was determined using two-tailed Student's t-test. *p < 0.05.

**Figure 2:**
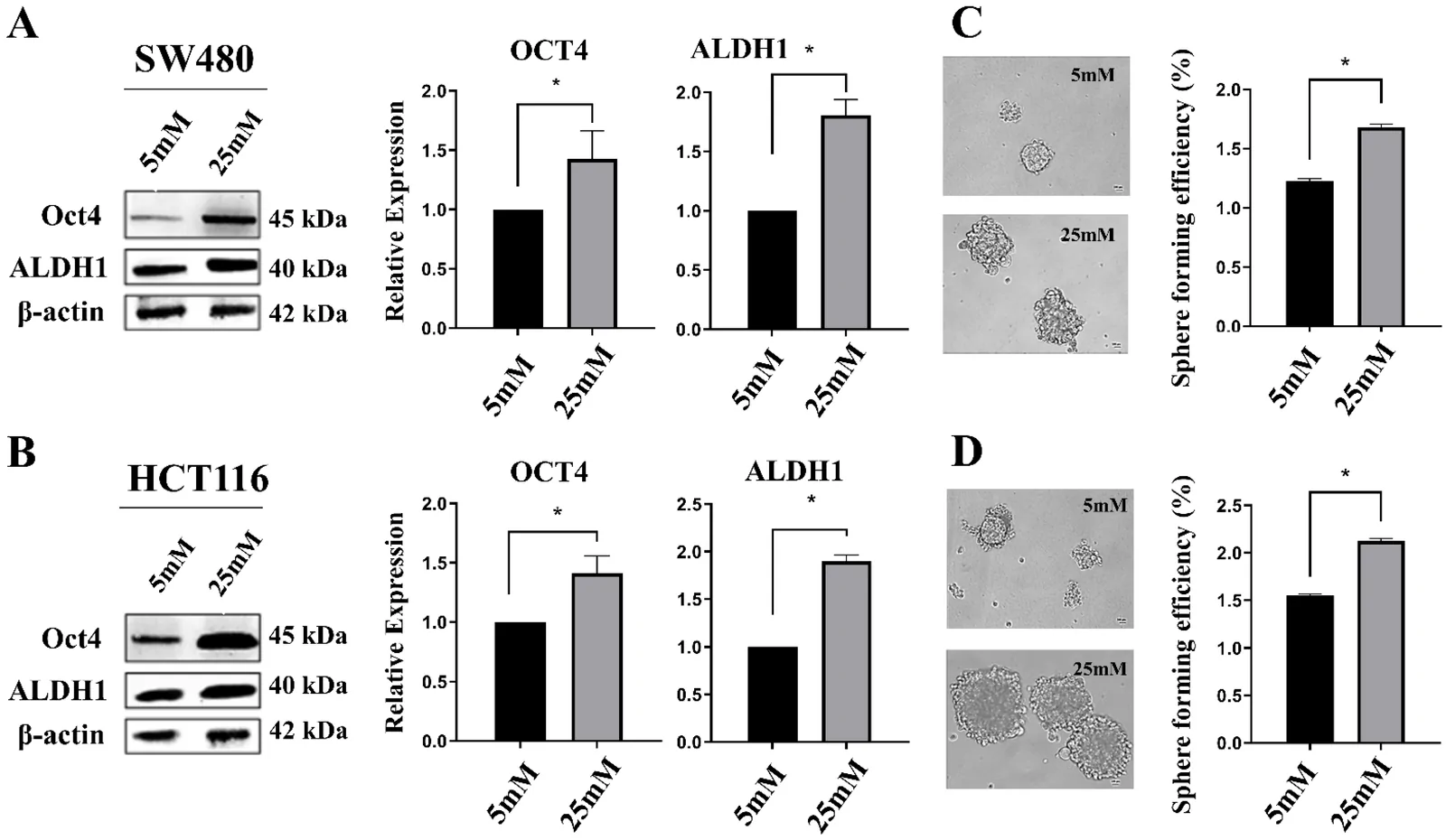
** High glucose promotes CSC-like properties in CRC cell lines.** Western blot analysis of CSC markers (OCT4 and ALDH1) in SW480 **(A)** and HCT116 **(B)** cells cultured under normal glucose (5 mM) or high glucose (25 mM) conditions. β-actin was used as a loading control. Sphere formation assays were performed to evaluate self-renewal capacity. Representative images of tumor spheres and quantification of sphere forming efficiency in SW480 **(C)** and HCT116 **(D)** cells are shown. Cells cultured in 25 mM glucose formed significantly more and larger spheres compared to those in 5 mM glucose. Data represent mean ± SD from three independent experiments (*p < 0.05). Statistical significance was determined using two-tailed Student's t-test. *p < 0.05.

**Figure 3:**
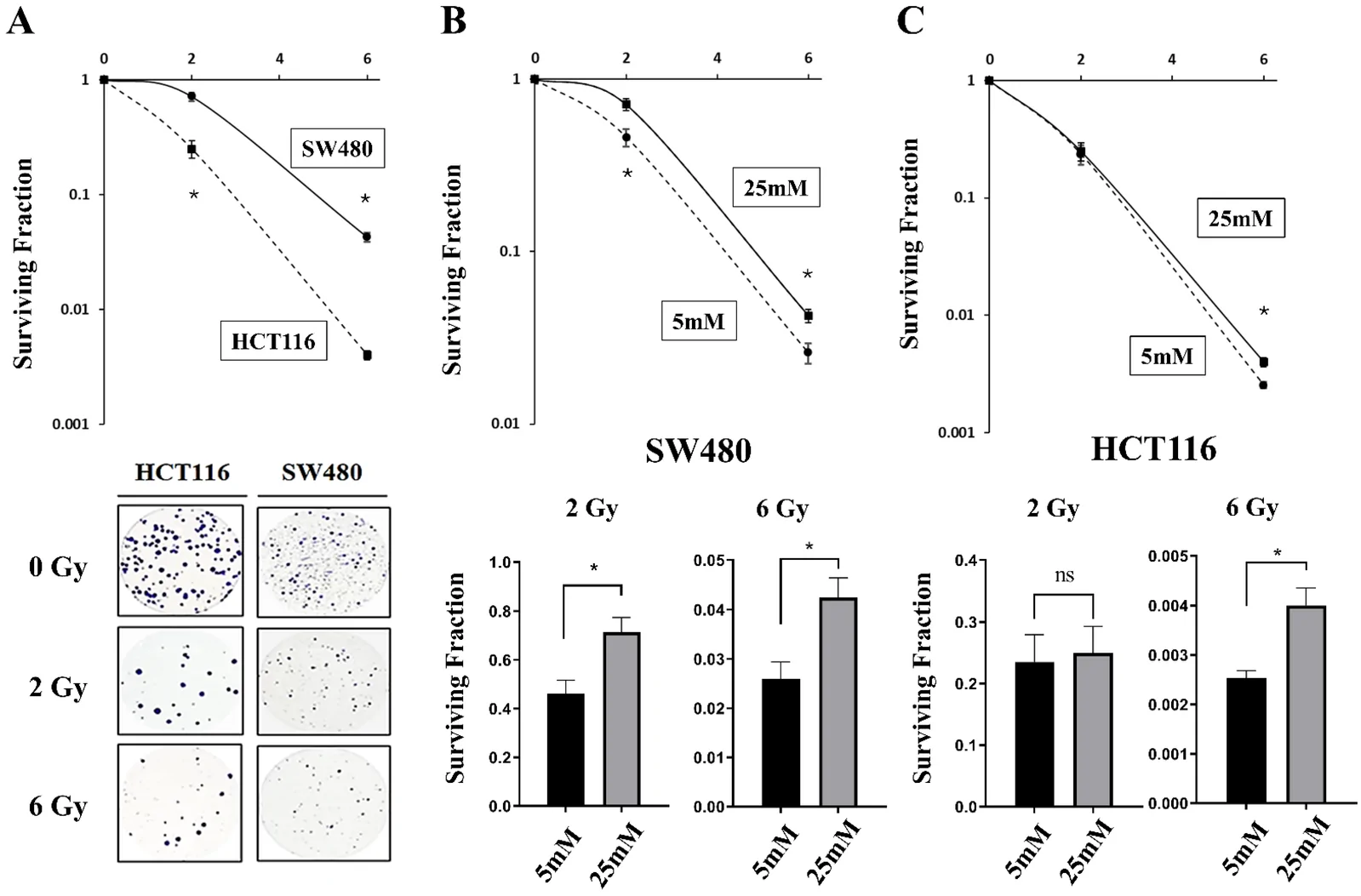
** High glucose microenvironment confers radioresistance to CRC cells.** Radiation clonogenic survival assays were performed to evaluate radiosensitivity. **(A)** Survival curves of SW480 and HCT116 cells following exposure to increasing doses of X-ray radiation (0, 2, 6 Gy), demonstrating the intrinsic radioresistance of SW480 cells compared to HCT116 cells. Clonogenic survival curves (top) and quantification of survival fractions at 2 Gy and 6 Gy (bottom) for SW480 **(B)** and HCT116 **(C)** cells cultured under normal glucose (NG, 5 mM) or high glucose (HG, 25 mM) conditions. Under HG conditions, SW480 cells showed significantly higher surviving fractions at both 2 Gy and 6 Gy, whereas HCT116 cells showed a significant increase at 6 Gy but not at 2 Gy. Data represent mean ± SD of triplicate determinations. Statistical significance was determined using two-tailed Student's t-test. *p < 0.05.

**Figure 4:**
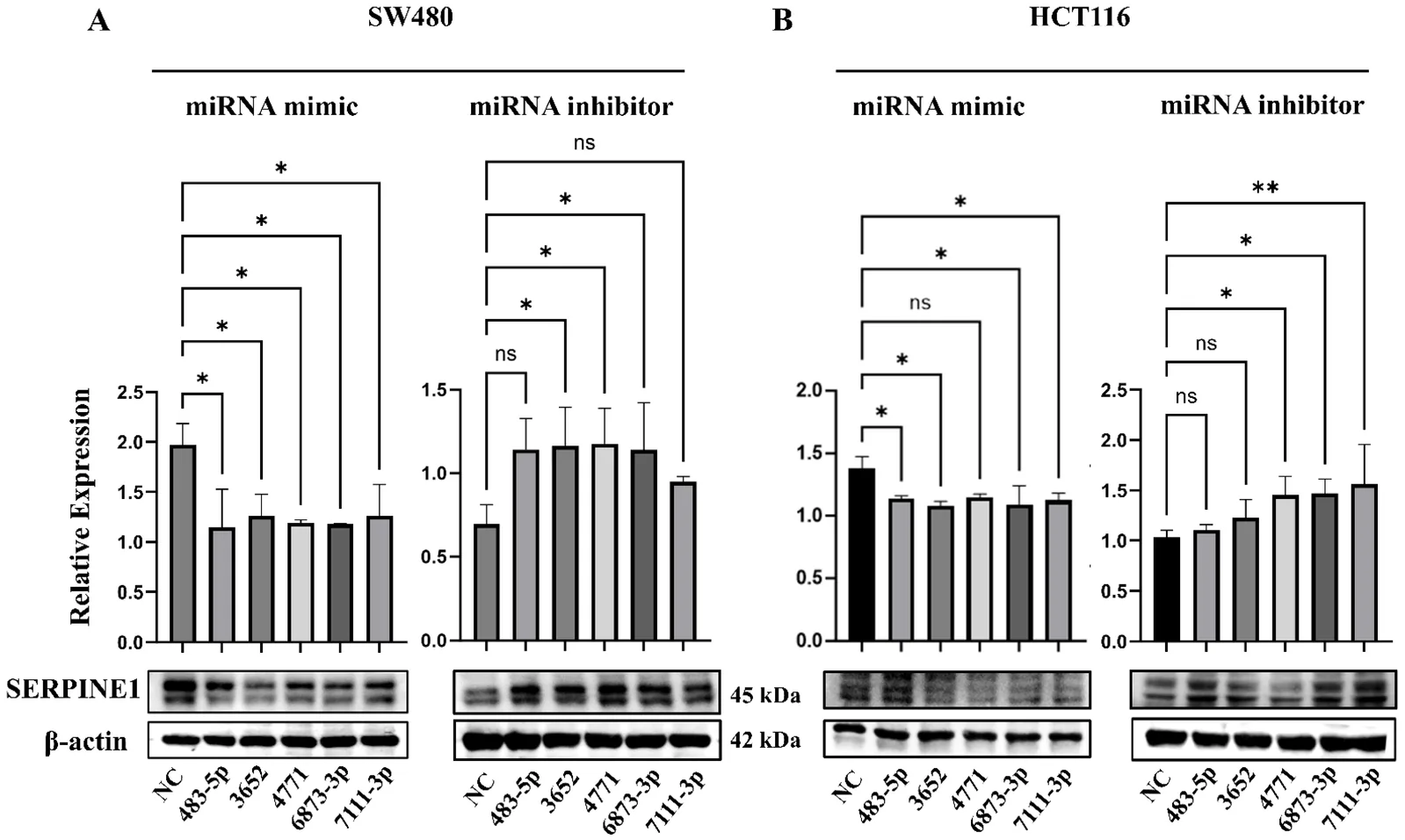
** Candidate hyperglycemia-associated miRNAs functionally regulate SERPINE1 expression in CRC cells.** Five hyperglycemia-associated downregulated exosomal miRNAs predicted to regulate SERPINE1 by TargetScan and miRDB were selected for mimic/inhibitor-based validation. SW480 (A) and HCT116 (B) cells were transfected with miR-483-5p, miR-3652, miR-4771, miR-6873-3p, or miR-7111-3p mimics or inhibitors, followed by Western blot analysis of SERPINE1 expression. β-actin served as the loading control. Quantified SERPINE1 protein levels were normalized to β-actin and expressed relative to the corresponding NC group. Data are presented as mean ± SD from at least three independent experiments. Statistical significance was determined using one-way ANOVA followed by Dunnett's post hoc test for multiple comparisons against the corresponding NC group. ns, not significant; *p < 0.05; **p < 0.01.

**Figure 5:**
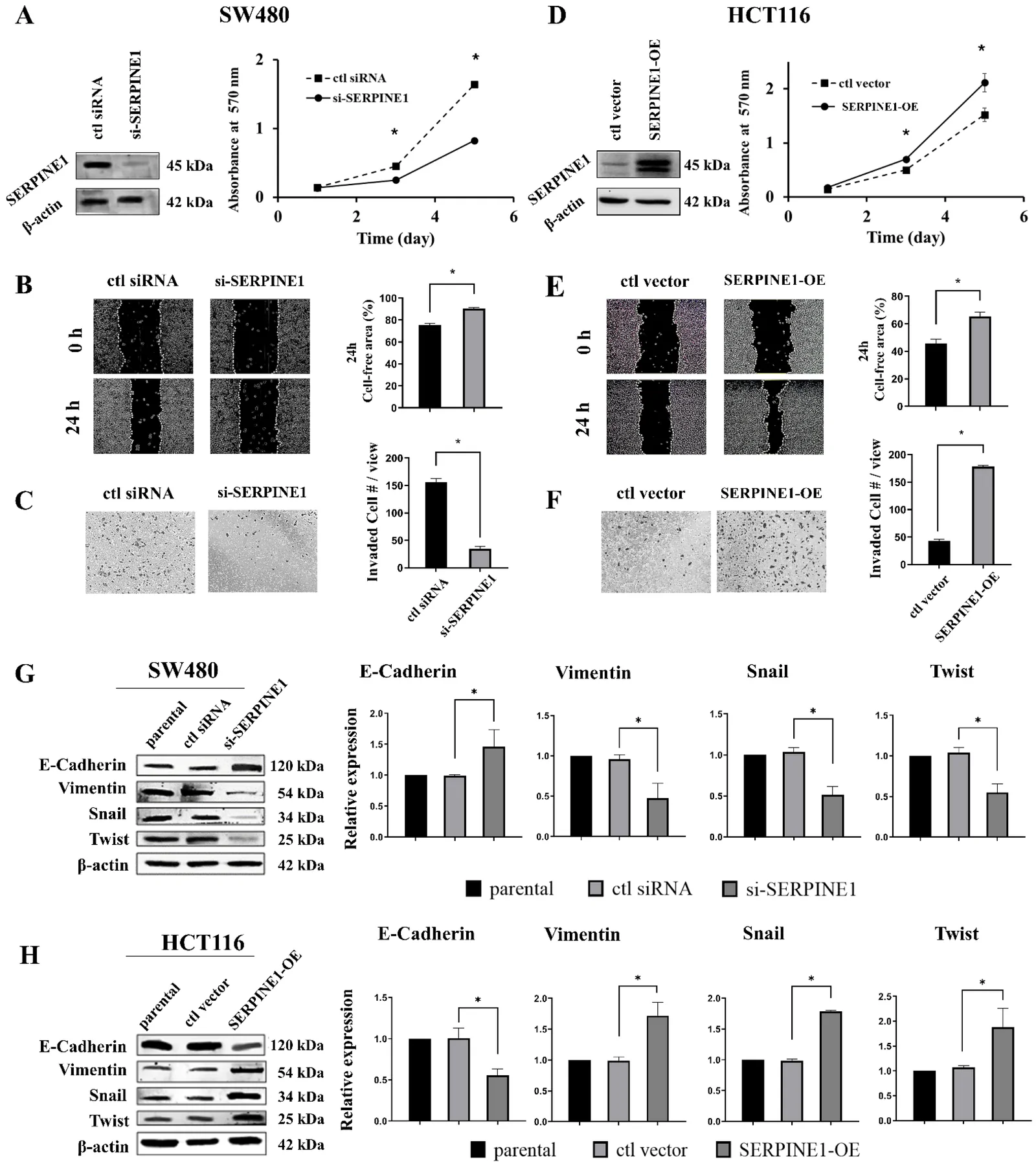
** SERPINE1 regulates malignant behaviors and EMT in CRC cells.** Loss- and gain-of-function studies were performed to validate the role of SERPINE1 under high-glucose conditions. SW480 cells were transfected with SERPINE1 siRNA or control siRNA, whereas HCT116 cells were transfected with a SERPINE1 overexpression plasmid or control vector. **(A)** Western blot analysis confirmed the knockdown efficiency of SERPINE1 in SW480 cells (left) and its effect on cell proliferation (MTT assay, right). Representative images and quantification of wound-healing migration **(B)** and Transwell invasion **(C)** assays in SERPINE1-knockdown (si-SERPINE1) vs. control (ctl siRNA) SW480 cells. **(D)** Western blot verification of SERPINE1 overexpression in HCT116 cells (left) and cell proliferation curves (right). Migration **(E)** and invasion **(F)** capabilities of SERPINE1-overexpressing (SERPINE1-OE) vs. vector control (ctl vector) HCT116 cells. Western blot analysis of EMT markers (E-cadherin, Vimentin, Snail, Twist) in SERPINE1-knockdown SW480 **(G)** and SERPINE1-overexpressing HCT116 **(H)** cells. β-actin was used as a loading control. Data represent mean ± SD from three independent experiments. For MTT cell proliferation, statistical significance was determined using two-way ANOVA followed by Tukey's post hoc test. For wound-healing and invasion, statistical significance was determined using two-tailed Student's t-test. For Western blot quantification, statistical significance was determined using one-way ANOVA followed by Dunnett's post hoc test. *p < 0.05.

**Figure 6:**
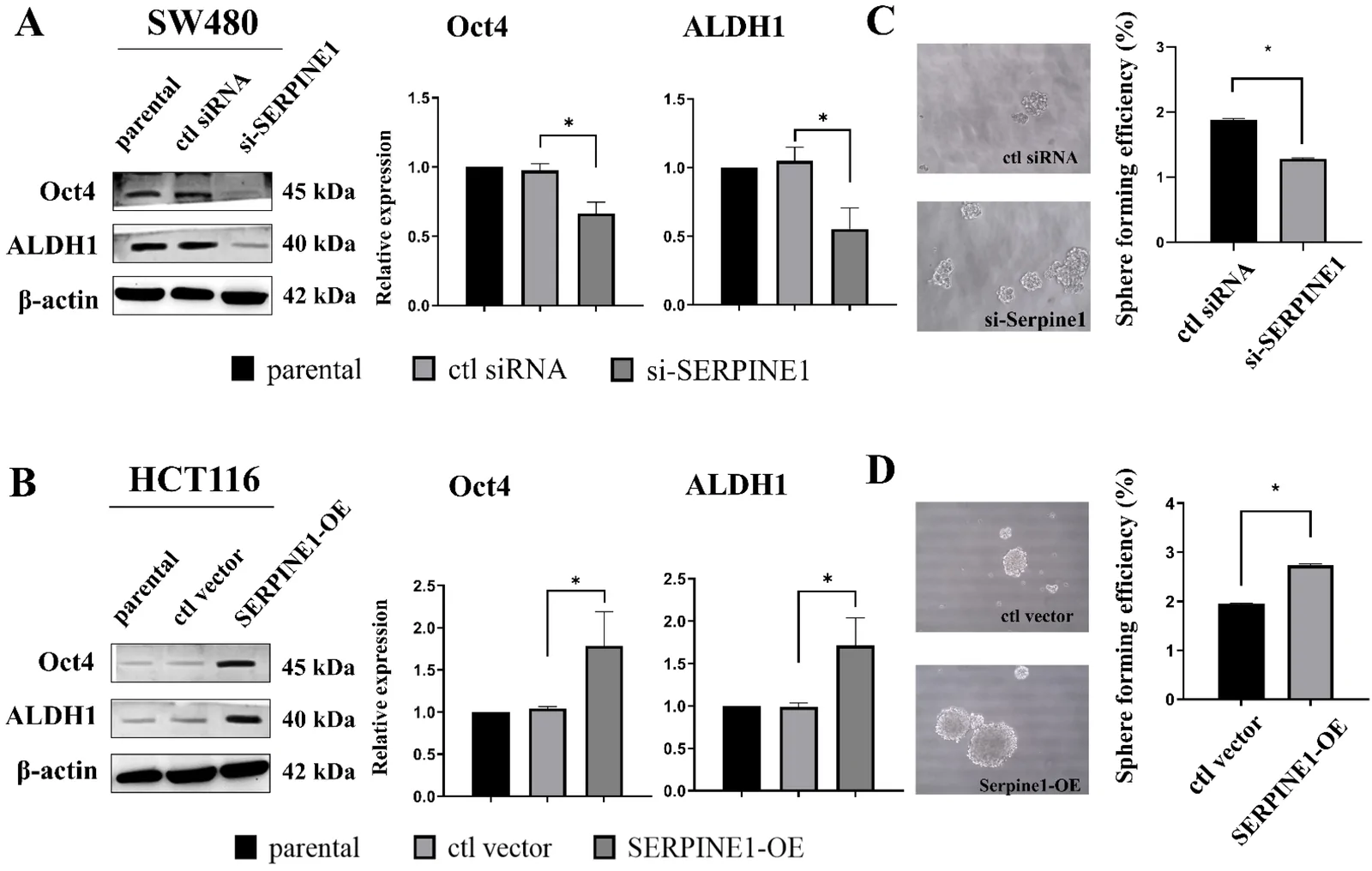
** SERPINE1 modulates CSC properties in CRC cells. (A, B)** Western blot analysis of stemness markers (OCT4 and ALDH1) in SERPINE1-knockdown (si-SERPINE1) SW480 cells **(A)** and SERPINE1-overexpressing (SERPINE1-OE) HCT116 cells **(B)** compared to their respective controls. β-actin served as a loading control. **(C, D)** Sphere formation assays were performed to evaluate self-renewal capacity. Representative images and quantification of sphere forming efficiency in SW480 **(C)** and HCT116 **(D)** cells are shown. Knockdown of SERPINE1 in SW480 cells significantly reduced the number and size of tumor spheres, whereas SERPINE1 overexpression in HCT116 cells enhanced sphere formation. All assays were performed under high-glucose conditions. Data represent mean ± SD from three independent experiments. For Western blot quantification, statistical significance was determined using one-way ANOVA followed by Dunnett's post hoc test. For sphere forming efficiency, statistical significance was determined using two-tailed Student's t-test. *p < 0.05.

**Figure 7:**
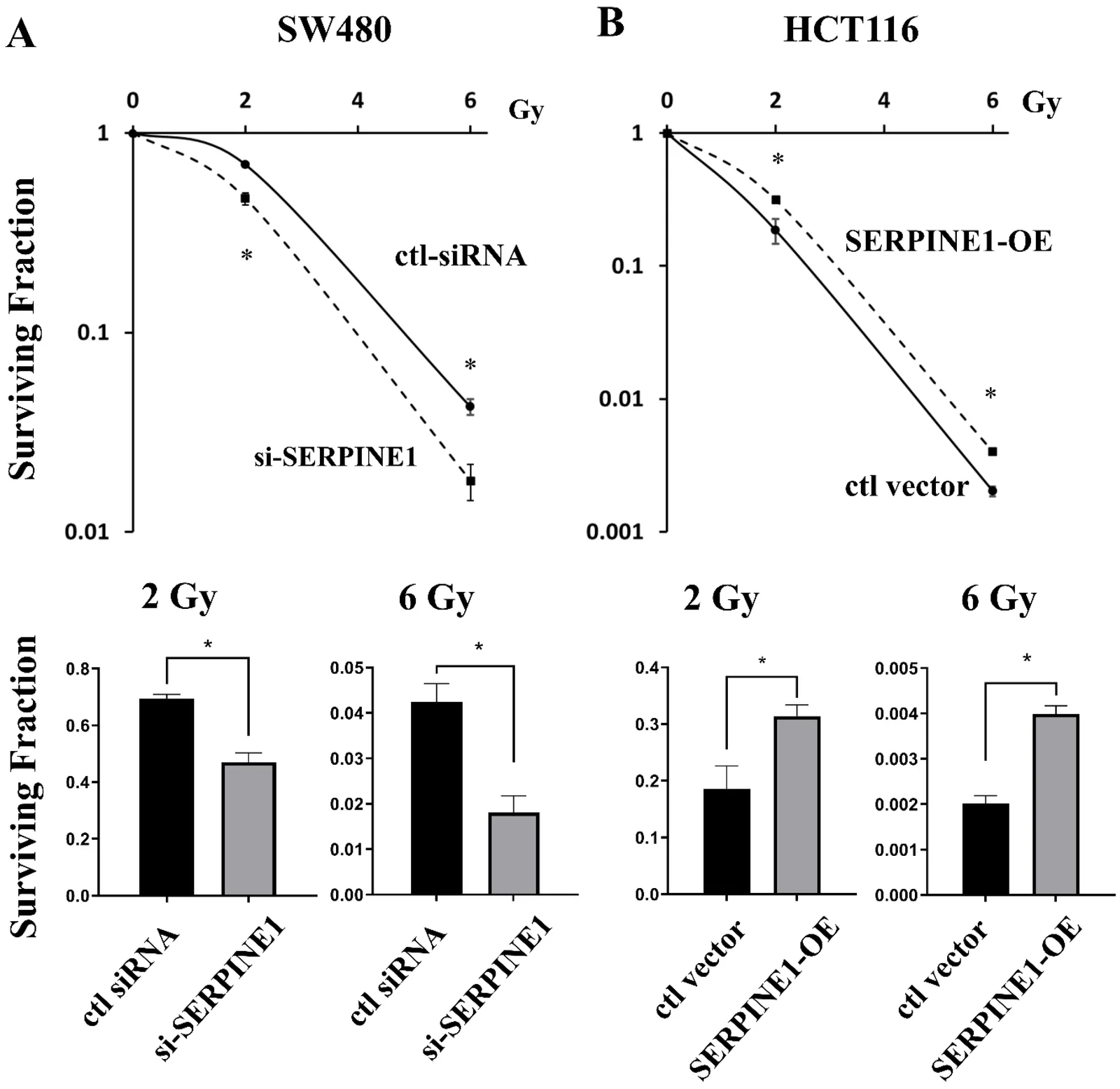
** SERPINE1 expression levels determine the radiosensitivity of CRC cells.** Clonogenic survival assays were performed under high-glucose conditions to assess the effect of SERPINE1 modulation on radiation sensitivity in SW480 and HCT116 cells. **(A)** Survival curves (top) and quantification of survival fractions at 2 Gy and 6 Gy (bottom) for SW480 cells transfected with control siRNA or SERPINE1 siRNA. Knockdown of SERPINE1 significantly sensitized SW480 cells to radiation. **(B)** Survival curves (top) and quantification of survival fractions (bottom) for HCT116 cells transfected with control vector or SERPINE1 overexpression plasmid (SERPINE1-OE). Overexpression of SERPINE1 conferred significant radioresistance to HCT116 cells. Data represent mean ± SD of triplicate determinations. Statistical significance was determined using two-tailed Student's t-test. *p < 0.05.

**Figure 8:**
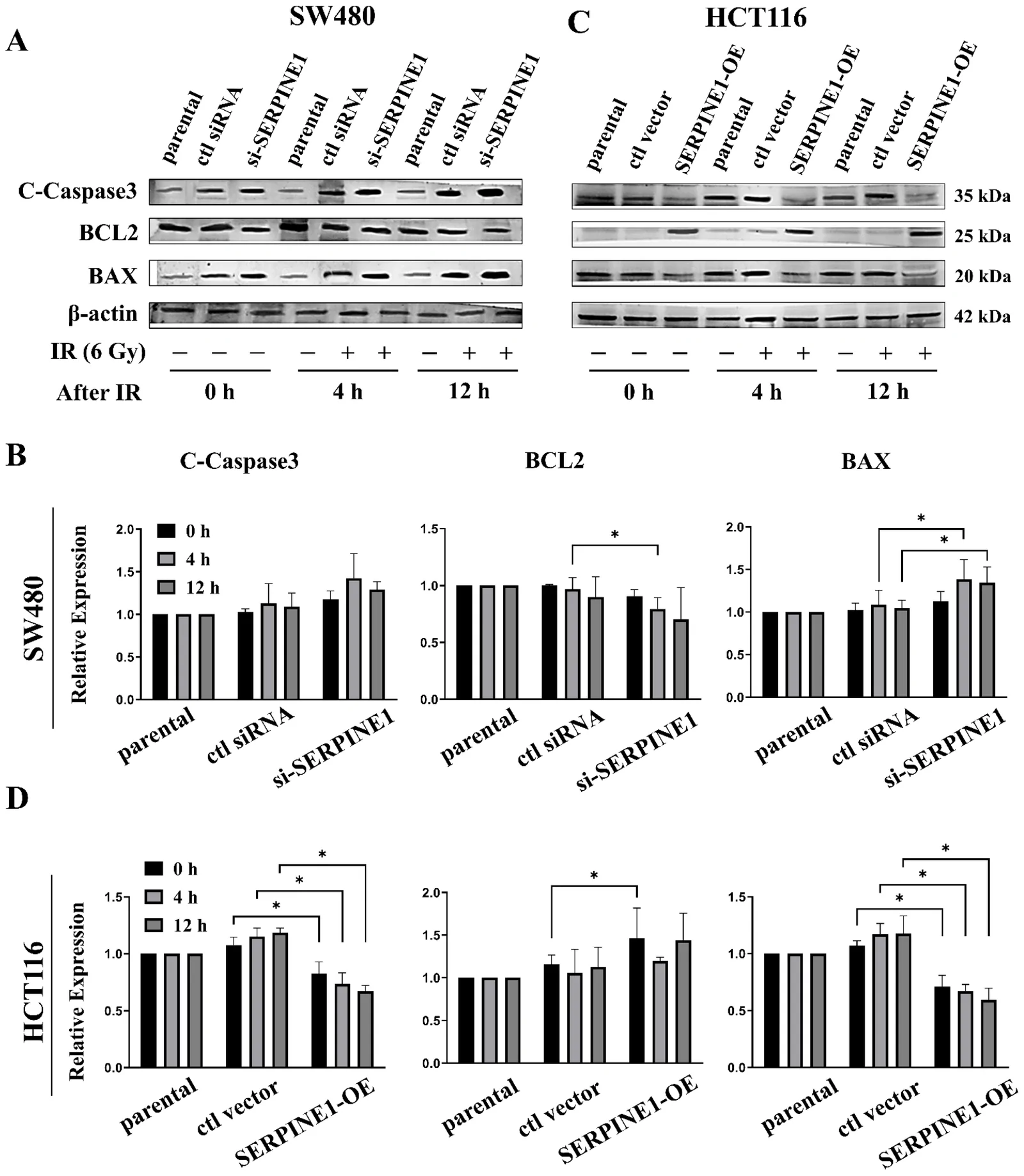
** SERPINE1 inhibits radiation-induced apoptosis in CRC cells.** Western blot analysis was performed under high-glucose conditions to examine the expression of apoptosis-related proteins following irradiation. **(A)** Representative Western blot images of cleaved-Caspase 3 (c-Caspase3), BCL2, and BAX in SW480 cells (parental, ctl siRNA, si-SERPINE1) harvested at 0, 4, and 12 h after 6 Gy irradiation. **(B)** Quantification of relative protein expression levels in SW480 cells. SERPINE1 knockdown significantly increased pro-apoptotic C-Caspase 3 and decreased anti-apoptotic BCL2 levels post-irradiation. **(C)** Representative Western blot images for HCT116 cells (parental, ctl vector, SERPINE1-OE) under the same experimental conditions. **(D)** Quantification of relative protein expression levels in HCT116 cells. SERPINE1 overexpression attenuated the radiation-induced increase in C-Caspase 3. β-actin served as a loading control. The images are representative of three independent experiments. Data represent mean ± SD from three independent experiments. Statistical significance was determined using two-way ANOVA followed by Tukey's post hoc test. *p < 0.05.

**Figure 9:**
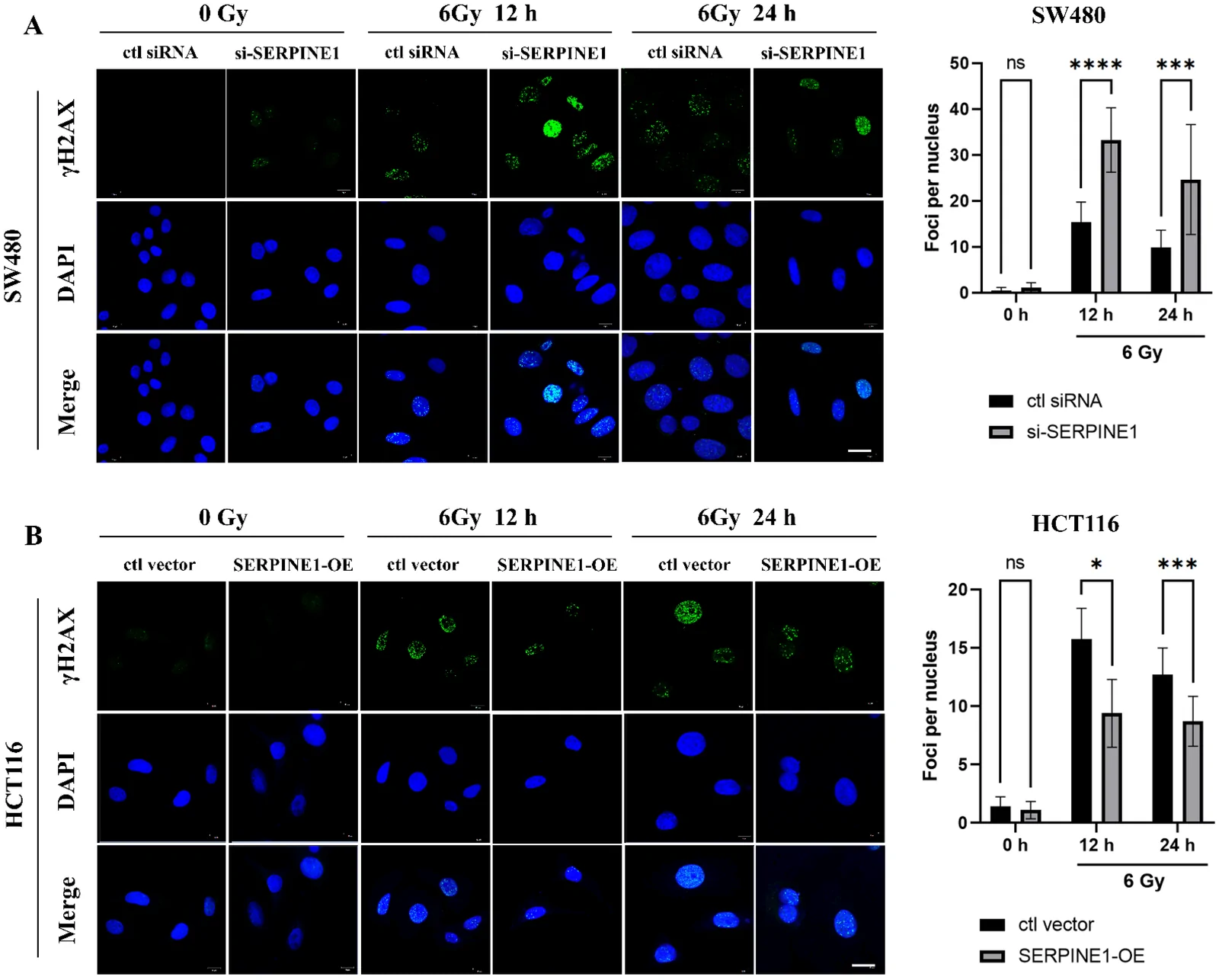
** SERPINE1 facilitates functional DNA repair resolution kinetics in CRC cells following irradiation.** Immunofluorescence analysis was performed under high-glucose conditions to evaluate the resolution kinetics of radiation-induced DNA DSBs by monitoring γ-H2AX foci. (A) Representative immunofluorescence images (left) and quantification (right) of γ-H2AX foci per nucleus in SW480 cells harvested without irradiation, and at 12, 24 h after 6 Gy irradiation. Under basal conditions (without irradiation), γ-H2AX foci levels remained low, whereas after irradiation, SERPINE1 knockdown significantly increased the number of residual γ-H2AX foci, indicating a delayed resolution of DNA damage. (B) Representative immunofluorescence images (left) and quantification (right) for HCT116 cells under the same experimental conditions. SERPINE1 overexpression significantly reduced residual γ-H2AX foci post-irradiation, indicating a more efficient resolution of DNA damage compared to vector controls. DAPI served as a nuclear counterstaining control. Scale bars = 10 μm. The images are representative of three independent experiments. Data represent mean ± SD from three independent experiments. Statistical significance was determined using two-way ANOVA followed by Tukey's post hoc test. *p < 0.05, ***p < 0.001, ****p < 0.0001; ns, non-significant.

**Figure 10:**
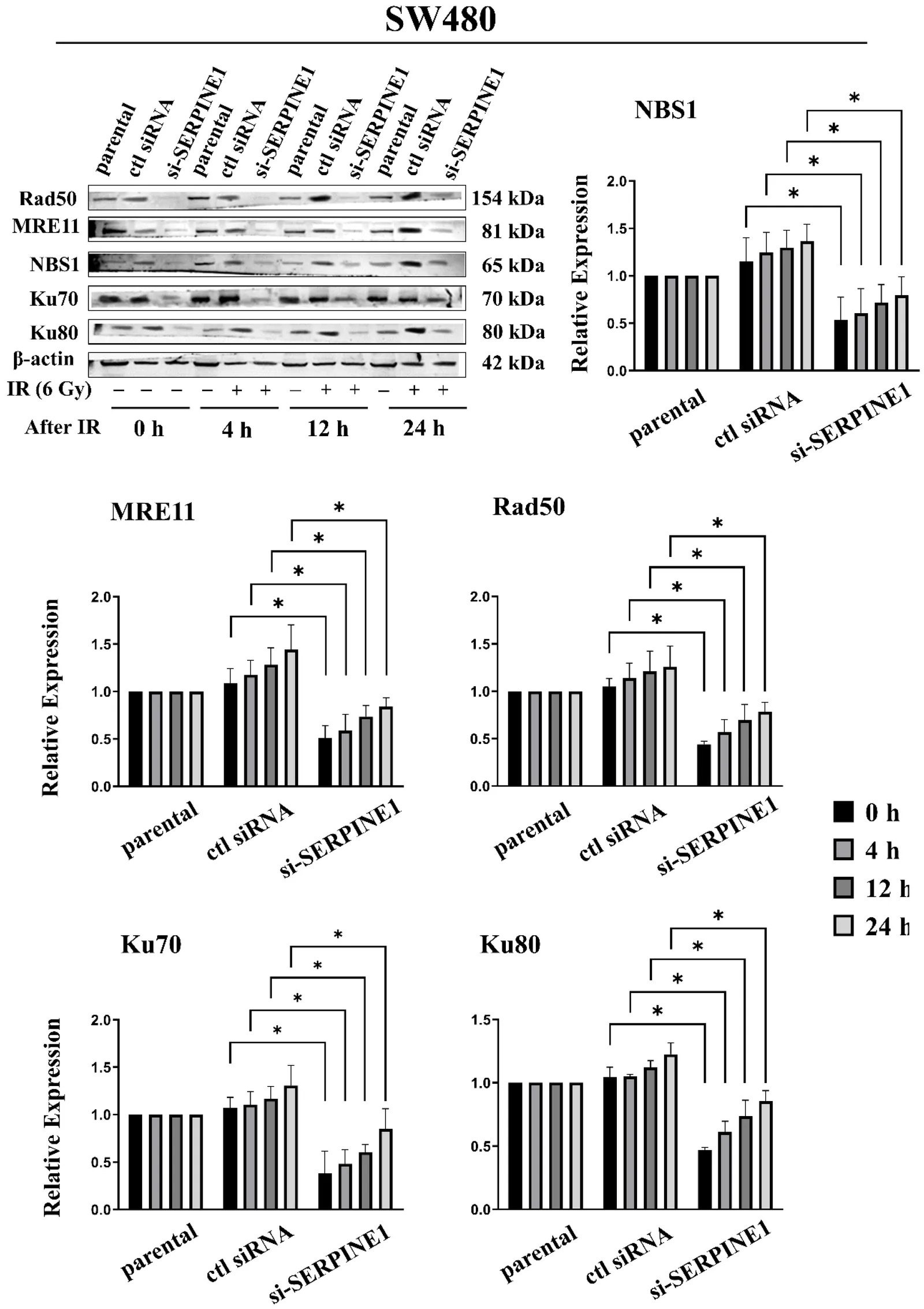
** SERPINE1 knockdown attenuates DNA repair-associated protein responses following irradiation in SW480 cells under high-glucose conditions.** Western blot analysis was performed to evaluate the expression of DNA double-strand break (DSB) repair-associated proteins in SERPINE1-knockdown SW480 cells and control siRNA-transfected cells under high-glucose conditions. Cells were harvested at 0, 4, 12, and 24 h after 6 Gy irradiation. Representative Western blot images and quantification of homologous recombination (HR)-associated MRN complex proteins, including RAD50, MRE11, and NBS1, and non-homologous end joining (NHEJ)-associated proteins, including Ku70 and Ku80, are shown. SERPINE1 knockdown reduced the baseline expression and/or post-irradiation induction of these DNA repair-associated proteins compared with control siRNA-transfected cells. β-actin served as a loading control. The images are representative of three independent experiments. Data are expressed as mean ± SD from three independent experiments. Statistical significance was determined using two-way ANOVA followed by Tukey's post hoc test. *p < 0.05.

**Figure 11:**
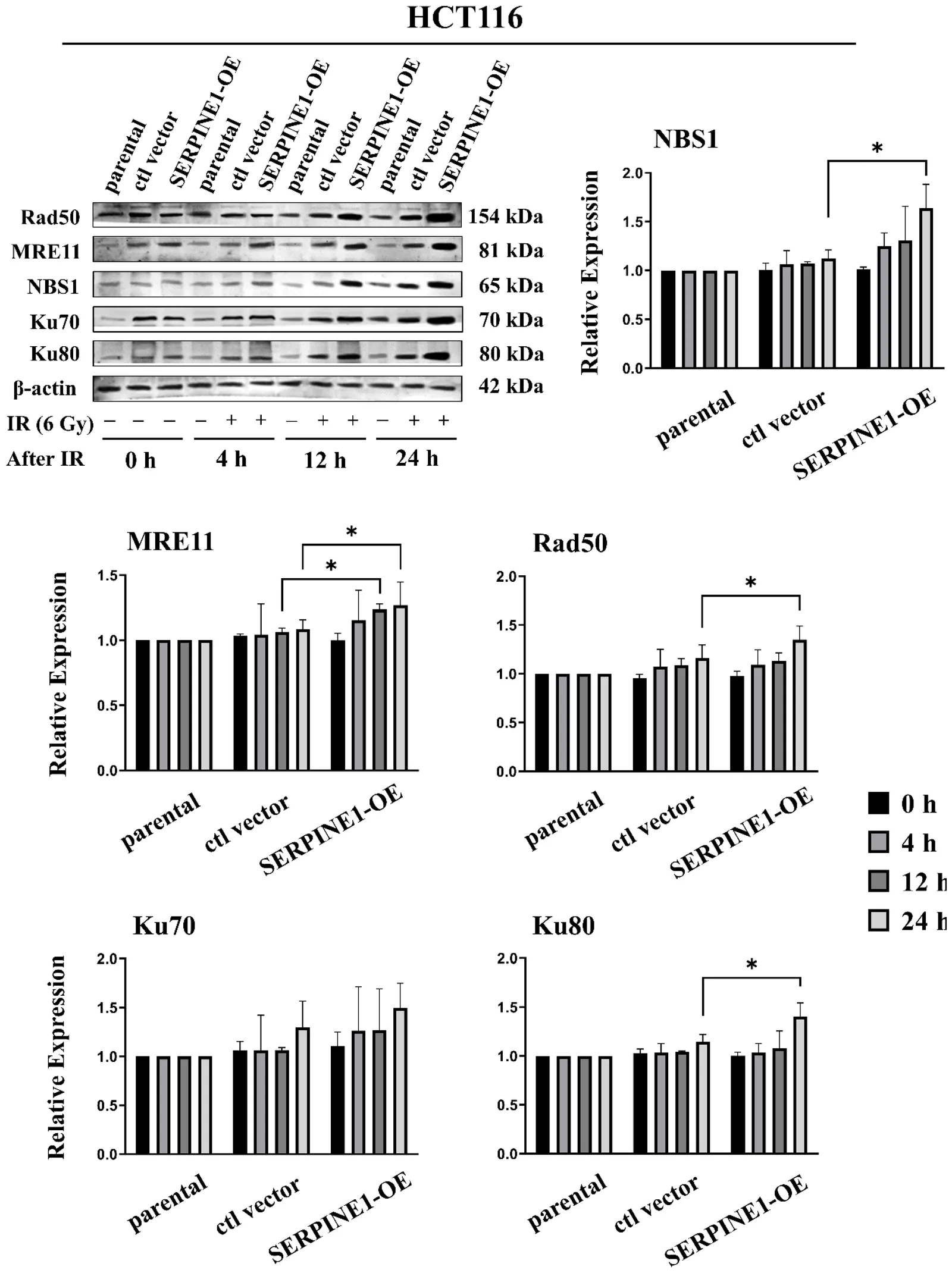
** SERPINE1 overexpression enhances DNA repair-associated protein responses following irradiation in HCT116 cells under high-glucose conditions.** Western blot analysis was performed to evaluate the expression of DNA double-strand break (DSB) repair-associated proteins in SERPINE1-overexpressing HCT116 cells and vector control cells under high-glucose conditions. Cells were harvested at 0, 4, 12, and 24 h after 6 Gy irradiation. Representative Western blot images and quantification of homologous recombination (HR)-associated MRN complex proteins, including RAD50, MRE11, and NBS1, and non-homologous end joining (NHEJ)-associated proteins, including Ku70 and Ku80, are shown. SERPINE1 overexpression increased the baseline expression and/or post-irradiation induction of these DNA repair-associated proteins compared with vector control cells. β-actin served as a loading control. The images are representative of three independent experiments. Data are expressed as mean ± SD from three independent experiments. Statistical significance was determined using two-way ANOVA followed by Tukey's post hoc test. *p < 0.05.

**Table 1:** 17 downregulated miRNAs in the hyperglycemia group with | normalized log2 fold change (log2FC) | ≥ 1.

miRNA	Normalized log2FC
hsa-miR-1224-5p	-5.34204
hsa-miR-3652	-5.34204
hsa-miR-7111-3p	-4.95502
hsa-miR-483-5p	-4.07889
hsa-miR-1247-5p	-3.54736
hsa-miR-1246	-3.29897
hsa-miR-9-3p	-2.83954
hsa-miR-320d	-2.56169
hsa-miR-6873-3p	-2.09785
hsa-miR-1290	-1.8101
hsa-miR-320b	-1.50175
hsa-miR-320c	-1.9353
hsa-miR-375-3p	-1.26959
hsa-miR-483-3p	-1.25458
hsa-miR-5189-3p	-1.25458
hsa-miR-1-3p	-1.25458
hsa-miR-4771	-1.01689
