# Supplementary material for: High-Glucose Environment Promotes Radioresistance in Colorectal Cancer via SERPINE1-Enhanced DNA Repair and Anti-Apoptosis
Source: Int J Med Sci. 2026 Sep 18;23(11):3356–73. doi: 10.7150/ijms.130794 (PMC13685041; doi:10.7150/ijms.130794)
Supplement: Supplementary file 1 [file ijmsv23p3356s1.pdf]

Figure S1

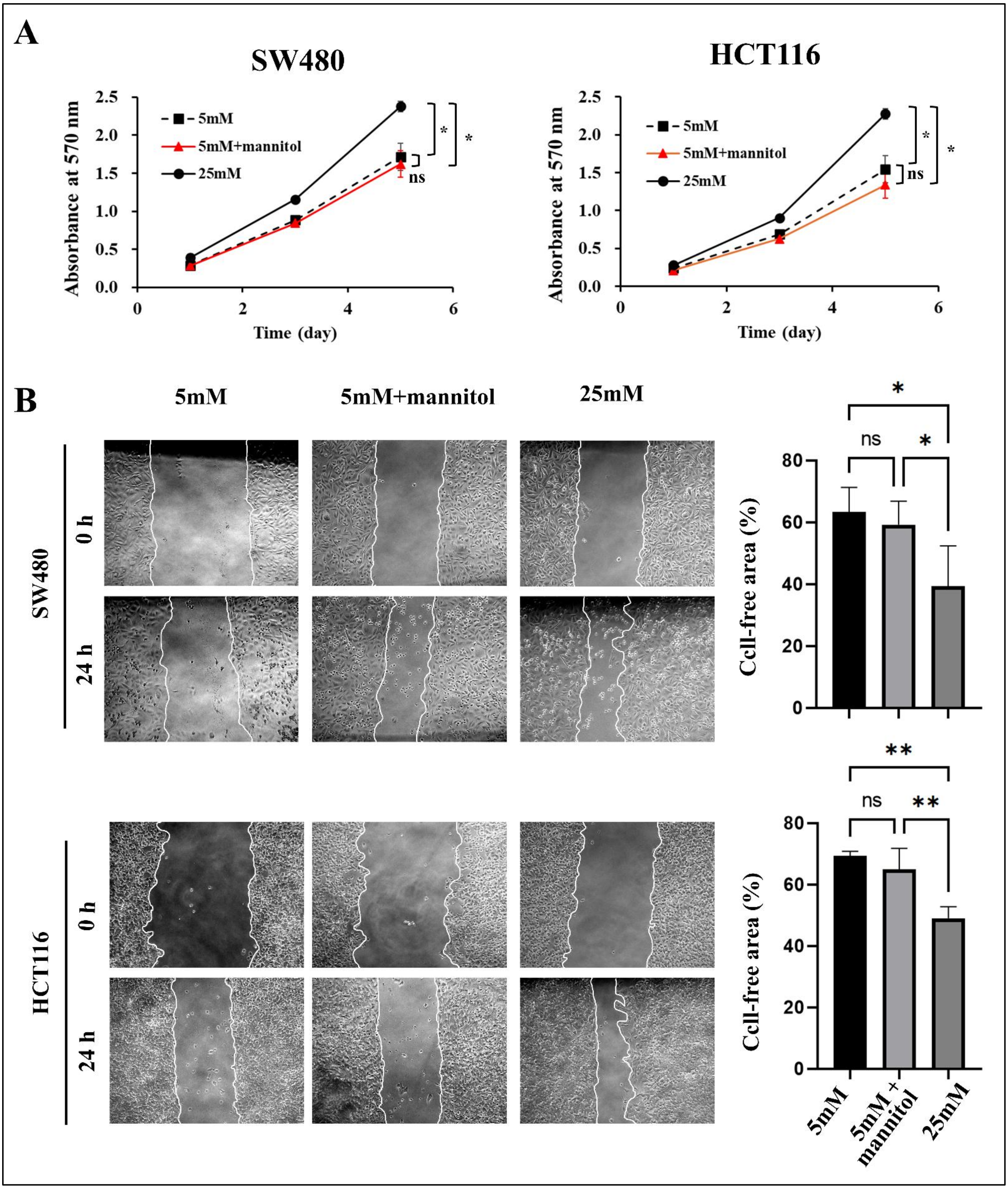

Figure S1. Mannitol osmotic control does not reproduce high glucose-induced proliferation and migration in CRC cells. SW480 and HCT116 cells were cultured under 5 mM glucose, 5 mM glucose supplemented with 20 mM mannitol, or 25 mM glucose conditions. (A) Cell proliferation was assessed using the MTT assay after culture for 1, 3, and 5 days in SW480 and HCT116 cells. (B) Cell migration was evaluated by wound-healing assay in SW480 and HCT116 cells. Representative images at 0 h and 24 h and

quantification of the remaining cell-free area are shown. The mannitol osmotic control did not significantly alter proliferation or wound closure compared with the 5 mM glucose group, whereas 25 mM glucose significantly enhanced both phenotypes. Data are expressed as mean ± S.D. from at least three independent experiments. Statistical significance was determined using one-way ANOVA followed by Tukey's post hoc test. ns, not significant; $^{*}P < 0.05$; $^{**}P < 0.01$.

Figure S2

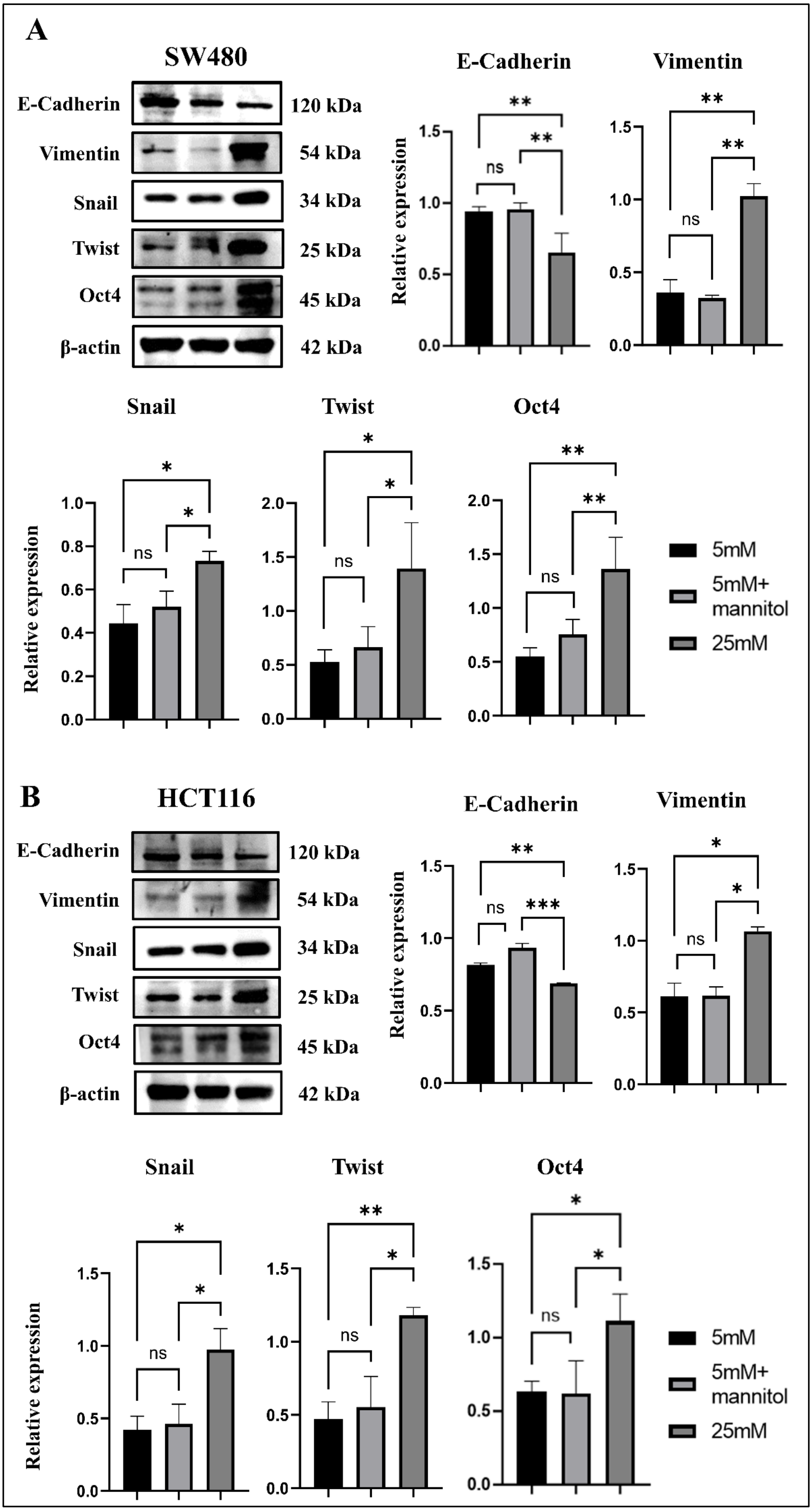

A
SW480
E-Cadherin
Vimentin
Snail
Twist
Oct4
β-actin
120 kDa
54 kDa
34 kDa
25 kDa
45 kDa
42 kDa
E-Cadherin
Vimentin
Snail
Twist
Oct4
Relative expression
ns
*
**
5mM
5mM+ mannitol
25mM
B
HCT116
E-Cadherin
Vimentin
Snail
Twist
Oct4
β-actin
120 kDa
54 kDa
34 kDa
25 kDa
45 kDa
42 kDa
E-Cadherin
Vimentin
Snail
Twist
Oct4
Relative expression
ns
*
**
***
5mM
5mM+ mannitol
25mM

Figure S2. Mannitol osmotic control does not reproduce high glucose-induced EMT and stemness marker expression in CRC cells. SW480 and HCT116 cells were cultured under 5 mM glucose, 5 mM glucose supplemented with 20 mM mannitol, or 25 mM glucose conditions. Western blot analysis was performed to examine the expression of EMT-related markers, including E-cadherin, Vimentin, Snail, and Twist, and the stemness-associated marker Oct4. Representative western blot images and quantification of relative protein expression are shown for SW480 (A) and HCT116 (B) cells. β-actin served as a loading control. The mannitol osmotic control did not reproduce the high glucose-induced downregulation of E-cadherin or upregulation of Vimentin, Snail, Twist, and Oct4. Data are expressed as mean ± S.D. from three independent experiments. Statistical significance was determined using one-way ANOVA followed by Tukey's post hoc test. ns, not significant; $*P < 0.05$; $**P < 0.01$.

Figure S3

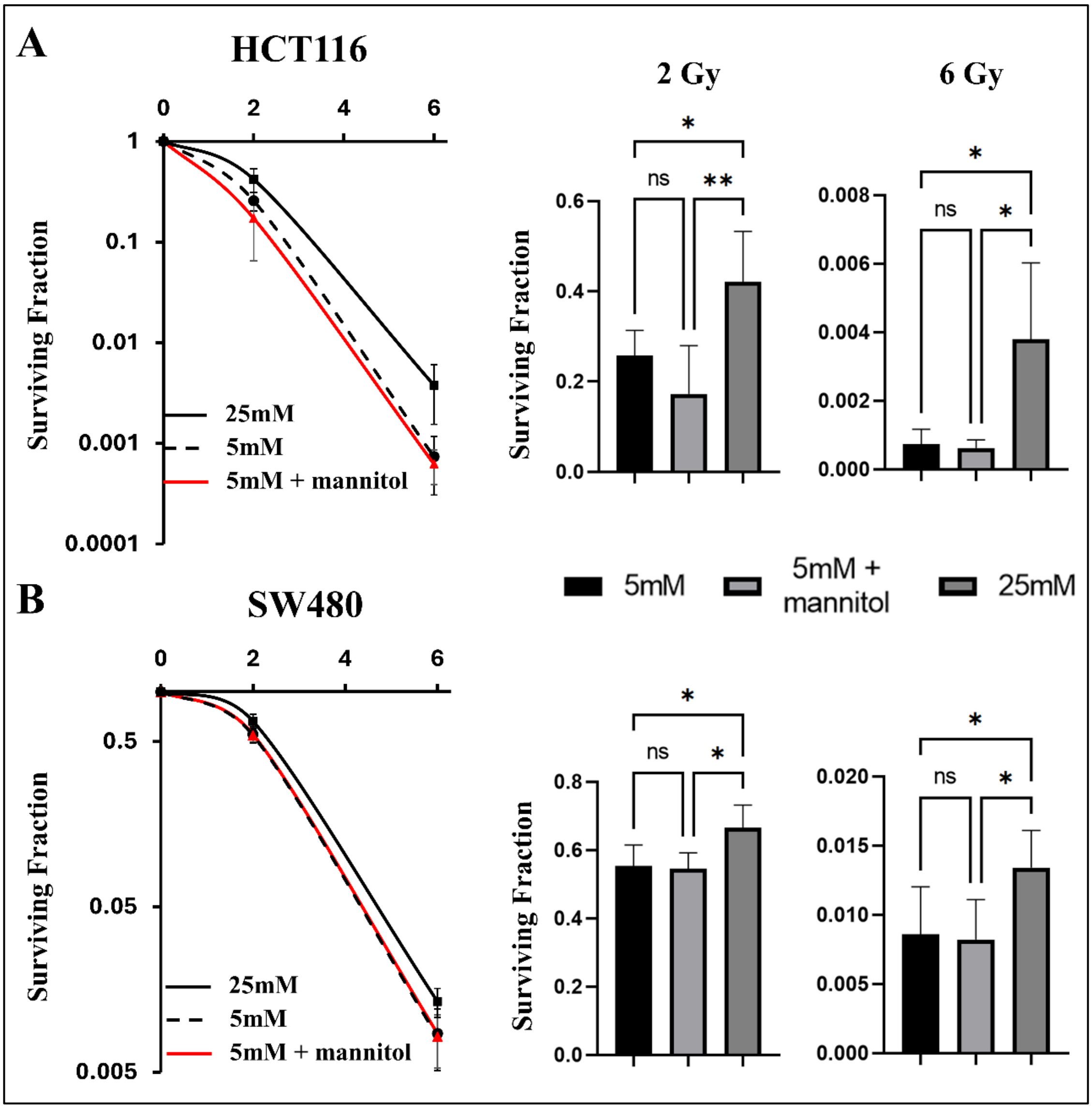

Figure S3. Mannitol osmotic control does not reproduce high glucose-induced clonogenic radioresistance in CRC cells. SW480 and HCT116 cells were cultured under 5 mM glucose, 5 mM glucose supplemented with 20 mM mannitol, or 25 mM glucose conditions, followed by X-ray irradiation at 0, 2, and 6 Gy. Clonogenic survival assays were performed to evaluate post-irradiation survival. Representative survival curves and quantification of surviving fractions are shown for HCT116 (A) and SW480 (B) cells. The 5 mM glucose plus mannitol group showed survival fractions comparable to the 5 mM glucose group, whereas cells cultured under 25 mM glucose exhibited significantly increased clonogenic survival after irradiation. These results indicate that high glucose-induced radioresistance was not reproduced by osmotic stress alone. Data are expressed as mean ± S.D. from three independent experiments. Statistical significance was determined using one-way ANOVA followed by Tukey's post hoc test. ns, not significant; *$P < 0.05$; **$P < 0.01$.

Figure S4.

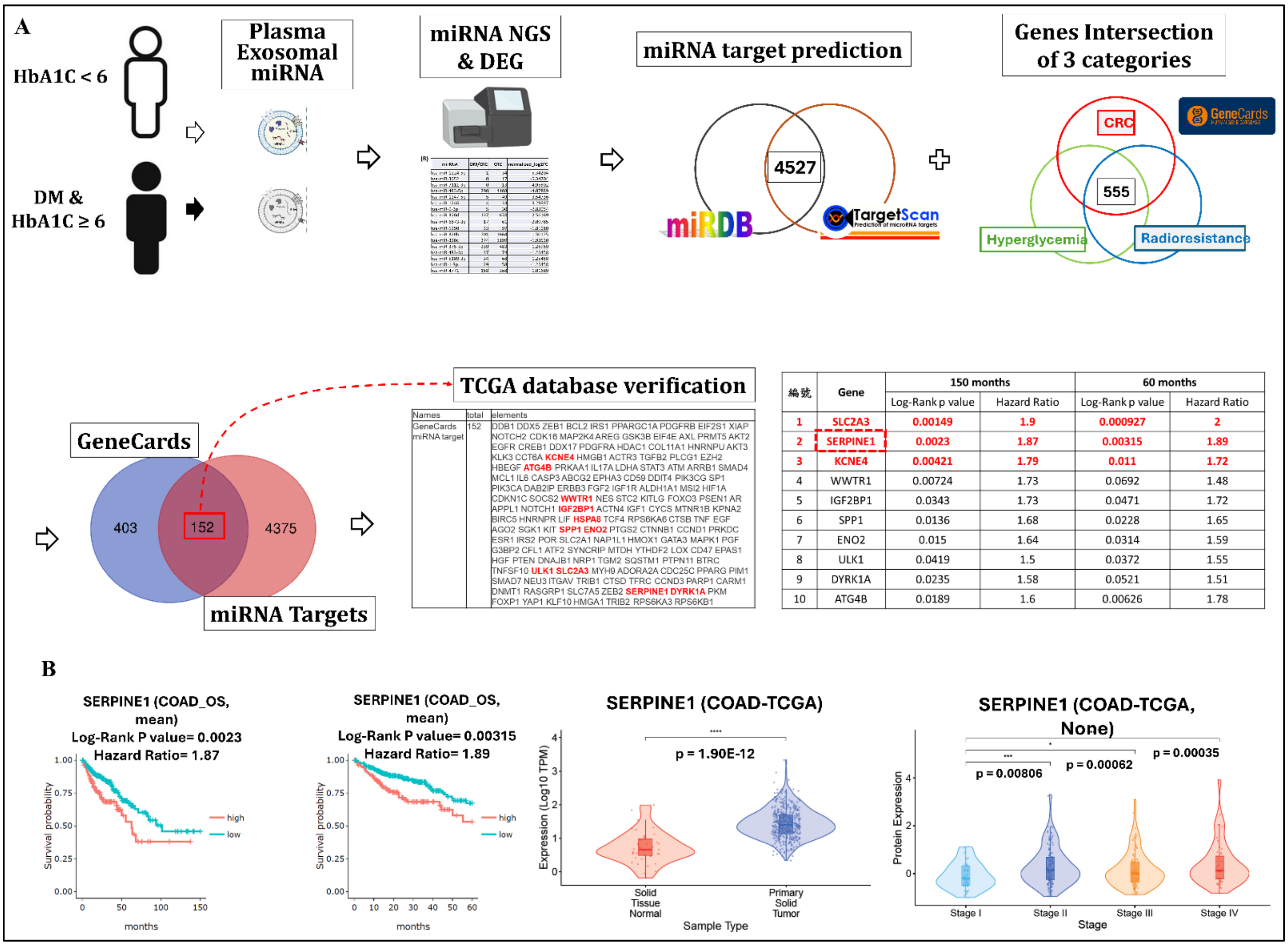

Figure S4. Integrated bioinformatics screening strategy identifies SERPINE1 as a key candidate gene. (A) A schematic overview of the multi-step screening process is shown. Relative miRNA expression changes were identified from pooled plasma exosomal miRNA profiles of hyperglycemic and normoglycemic CRC patients. Potential target genes of downregulated miRNAs were predicted using miRDB and TargetScan and intersected with disease-related gene sets ("Colorectal Cancer", "Hyperglycemia", and "Radioresistance") from GeneCards, yielding 152 candidate genes. These candidates were further validated using the TCGA dataset; the table lists the top 10 genes ranked by clinical prognostic significance (Log-Rank *P* value and Hazard Ratio). (B) Kaplan-Meier overall survival curves for SERPINE1 (High vs. Low expression) and its expression levels in tumor versus normal tissues and across tumor stages are shown.

Table S1. Sequences of miRNA mimics and inhibitors used for functional validation

miRNA	Accession No.	mimic sequence (5′→3′)	inhibitor sequence (5′→3′)
miR-483-5p	MIMAT0004761	AAGACGGGAGGAAAGAAGGGAG	CUCCCUUCUUUCCUCCCGUCUU
miR-3652	MIMAT0018072	CGGCUGGAGGUGUGAGGA	UCCUCACACCUCCAGCCG
miR-4771	MIMAT0019925	AGCAGACUUGACCUACAAUUA	UAAUUGUAGGUCAAGUCUGCU
miR-6873-3p	MIMAT0027647	UUCUCUCUGUCUUUCUCUCUCAG	CUGAGAGAGAAAGACAGAGAGAA
miR-7111-3p	MIMAT0028120	AUCCUCUCUUCCCUCCUCCCAG	CUGGGAGGAGGGAAGAGAGGAU
